# The integrated structure of care: evidence for the efficacy of models of clinical governance in the prevention of fragility fractures after recent sentinel fracture after the age of 50 years

**DOI:** 10.1007/s11657-023-01316-9

**Published:** 2023-08-21

**Authors:** L. Cianferotti, G. Porcu, R. Ronco, G. Adami, R. Alvaro, R. Bogini, A. P. Caputi, B. Frediani, D. Gatti, S. Gonnelli, G. Iolascon, A. Lenzi, S. Leone, R. Michieli, S. Migliaccio, T. Nicoletti, M. Paoletta, A. Pennini, E. Piccirilli, M. Rossini, U. Tarantino, M. L. Brandi, G. Corrao, A. Biffi

**Affiliations:** 1 Italian Bone Disease Research Foundation (FIRMO), Florence, Italy; 2 Department of Statistics and Quantitative Methods, National Centre for Healthcare Research and Pharmacoepidemiology, University of Milano-Bicocca, Milan, Italy; 3 Unit of Biostatistics, Epidemiology, and Public Health, Department of Statistics and Quantitative Methods, University of Milano-Bicocca, Milan, Italy; 4 Rheumatology Unit, University of Verona, Verona, Italy; 5 Department of Biomedicine and Prevention, University of Rome Tor Vergata, Rome, Italy; 6 Local Health Unit (USL) Umbria, Perugia, Italy; 7 Department of Pharmacology, School of Medicine, University of Messina, Sicily, Italy; 8 Department of Medicine, Surgery and Neurosciences, Rheumatology Unit, University of Siena, Azienda Ospedaliero-Universitaria Senese, Siena, Italy; 9 Department of Medicine, Surgery and Neuroscience, Policlinico Le Scotte, University of Siena, Siena, Italy; 10 Department of Medical and Surgical Specialties and Dentistry, University of Campania “Luigi Vanvitelli”, Naples, Italy; 11 Department of Experimental Medicine, Sapienza University of Rome, Viale del Policlinico, Rome, Italy; 12 AMICI Onlus, Associazione nazionale per le Malattie Infiammatorie Croniche dell’Intestino, Milan, Italy; 13 Italian Society of General Medicine and Primary Care (SIMG), Florence, Italy; 14 Department of Movement, Human and Health Sciences, Foro Italico University, Rome, Italy; 15 CnAMC, Coordinamento nazionale delle Associazioni dei Malati Cronici e rari di Cittadinanzattiva, Rome, Italy; 16 Department of Clinical Sciences and Translational Medicine, University of Rome “Tor Vergata”, Rome, Italy; 17 Department of Orthopedics and Traumatology, “Policlinico Tor Vergata” Foundation, Rome, Italy

**Keywords:** Fracture liaison service, Fragility fracture, Secondary prevention, Systematic review

## Abstract

***Summary*:**

Randomized clinical trials and observational studies on the implementation of clinical governance models, in patients who had experienced a fragility fracture, were examined. Literature was systematically reviewed and summarized by a panel of experts who formulated recommendations for the Italian guideline.

**Purpose:**

After experiencing a fracture, several strategies may be adopted to reduce the risk of recurrent fragility fractures and associated morbidity and mortality. Clinical governance models, such as the fracture liaison service (FLS), have been introduced for the identification, treatment, and monitoring of patients with secondary fragility fractures. A systematic review was conducted to evaluate the association between multidisciplinary care systems and several outcomes in patients with a fragility fracture in the context of the development of the Italian Guidelines.

**Methods:**

PubMed, Embase, and the Cochrane Library were investigated up to December 2020 to update the search of the Scottish Intercollegiate Guidelines Network. Randomized clinical trials (RCTs) and observational studies that analyzed clinical governance models in patients who had experienced a fragility fracture were eligible. Three authors independently extracted data and appraised the risk of bias in the included studies. The quality of evidence was assessed using the Grading of Recommendations Assessment, Development, and Evaluation methodology. Effect sizes were pooled in a meta-analysis using random-effects models. Primary outcomes were bone mineral density values, antiosteoporotic therapy initiation, adherence to antiosteoporotic medications, subsequent fracture, and mortality risk, while secondary outcomes were quality of life and physical performance.

**Results:**

Fifteen RCTs and 62 observational studies, ranging from very low to low quality for bone mineral density values, antiosteoporotic initiation, adherence to antiosteoporotic medications, subsequent fracture, mortality, met our inclusion criteria. The implementation of clinical governance models compared to their pre-implementation or standard care/non-attenders significantly improved BMD testing rate, and increased the number of patients who initiated antiosteoporotic therapy and enhanced their adherence to the medications. Moreover, the treatment by clinical governance model respect to standard care/non-attenders significantly reduced the risk of subsequent fracture and mortality. The integrated structure of care enhanced the quality of life and physical function among patients with fragility fractures.

**Conclusions:**

Based on our findings, clinicians should promote the management of patients experiencing a fragility fracture through structured and integrated models of care. The task force has formulated appropriate recommendations on the implementation of multidisciplinary care systems in patients with, or at risk of, fragility fractures.

**Supplementary Information:**

The online version contains supplementary material available at 10.1007/s11657-023-01316-9.

## Introduction

Fragility fractures impose a massive burden on health care systems and the global community [1]. Such fractures are the hallmark of osteoporosis, causing high morbidity, loss of independence, and negatively affecting the quality of life [2]. People who have experienced a fragility fracture (i.e., spontaneous or low-traumatic) have a greater fracture risk immediately after the event [3]. However, antiosteoporotic therapy administered soon after a fragility fracture may mitigate this risk [4]. Unfortunately, temporary or permanent medication discontinuation are frequent (> 50–80%), especially in secondary prevention [5, 6].

The detection of a major fragility fracture (i.e., spontaneous fracture or fracture resulting from a low-impact trauma/fall from standing height or less, occurring at the vertebral bodies, proximal hip, wrist, or humerus) is crucial to identify patients at high risk of subsequent fractures, evaluate bone fragility, and prescribe antiosteoporotic medication [7]. Following an initial fracture, several strategies may be adopted, although secondary preventive measures might not be promptly used. In the last decade, several initiatives (at the various levels—local, regional, national, and international) have been undertaken to improve secondary fracture prevention; these include fracture liaison services (FLS). FLS models were originally introduced in the orthopedic departments of tertiary referral centers as multidisciplinary teams, including coordinators, orthopedic surgeons, bone nurses, bone doctors (internists, endocrinologists, orthogeriatrics, rheumatologists), radiologists, and physiatrists, centered on the fractured patient. At present, these programs also involve primary care in the form of the general practitioner, which is fundamental to promote and support short- and long-term adherence to the antiosteoporotic treatments [8]. These models have proven to be effective in different settings and clinical pathways [9]. Indeed, FLS programs have been demonstrated to reduce fracture-related morbidity and mortality as well as decrease healthcare costs for the secondary prevention of fragility fractures [10].

This systematic review and meta-analysis aims to provide recommendations based on the best available evidence on the efficacy and effectiveness of clinical governance models. The findings may support decision-makers to minimize the cost and social burden associated with fragility fractures.

## Methods

We conducted a systematic review to support the Panel of the Italian Fragility Fracture Guidelines (published on the platform of the Italian National Institute of Health) in formulating recommendations. Adopting the GRADE-ADOLOPMENT methodology [11] and the standards defined by the *Sistema Nazionale Linee Guida* (SNLG [12]), the multidisciplinary panel updated the clinical question of the Scottish guidelines (SIGN, Scottish Intercollegiate Guidelines Network [13]): “Is the use of clinical governance models, such as the so-called fracture liaison services, suitable for the post-fracture patient’s management?”

### Inclusion and exclusion criteria

Randomized clinical trials (RCTs) and/or observational studies were selected if they met the following criteria: (1) population: patients who experienced a fragility fracture; (2) intervention: clinical governance models, such as case manager interventions or FLS; (3) comparison: standard care; (4) outcome: (i) primary outcome measures, specifically bone mineral density (BMD) testing rate, antiosteoporotic therapy initiation, adherence to antiosteoporotic medications, subsequent fracture, and mortality risk, and (ii) secondary outcomes were quality of life and physical performance.

Studies were excluded if they (i) were not published in the English language, (ii) did not report original findings (i.e., letters, case report), (iii) did not identify patients affected by a fragility fracture, or (iv) were not before and after studies on the clinical governance model implementation or did not consider standard treatment/non-attenders/another model as a comparator.

### Data source and search strategy

We performed a PubMed, Embase, and Cochrane Library search to update the search of the SIGN guidelines, from 2013 up to 17 December 2020, and identified publications on clinical governance models for patients who have sustained a fragility fracture. A systematic review of the available literature was carried out according to the Preferred Reporting Items for Systematic Reviews and Meta-analyses (PRISMA) [14] (Supplemental Material, Table S1). The search strategy (Supplemental Material, Table S2) included specific keywords and/or corresponding MeSH terms related to “fragility fracture” AND “integrated models of care.” We checked the reference lists of the studies and the systematic reviews identified during the search process.

### Study selection and data extraction

Three independent authors (AB, GP, RR) screened titles and abstracts according to the search strategy and then assessed the full text of the potentially relevant studies. Discrepancies between reviewers were resolved by a consensus meeting. For each included publication, the following information was extracted: (i) first author, year, and country of publication, (ii) study setting, (iii) type of population, (iv) intervention and comparator, and (v) follow-up period.

### Quality of studies

The systematic reviews were evaluated using the AMSTAR-2 checklist [15]. The quality of each included publication, derived by our search, was assessed using the Cochrane Risk of Bias (RoB) tool for RCTs [16] and the Newcastle-Ottawa scales [17] for observational studies. The following domains of the Cochrane RoB tool were appraised: selection bias (random sequence generation and allocation concealment), performance bias (blinding of participants and personnel), detection bias (blinding of outcome assessment), attrition bias (incomplete outcome data), reporting bias (selective reporting), and other bias (such as funding bias). Each domain was classified as “high,” “low,” or “unclear” RoB to assess to what extent the publication did not provide sufficient information. In the Newcastle-Ottawa scales, the following domains were evaluated: selection, comparability, and outcome. The threshold for identifying high-quality studies was more than five points.

### Quality of evidence

The quality of evidence of each outcome was judged by evaluating five dimensions (risk of bias, consistency of effect, imprecision, indirectness, and publication bias) using the Grading of Recommendations Assessment Development and Evaluation (GRADE) approach [18]. The evidence was downgraded from “high quality” by one level if serious limitations were found for each of the five dimensions, or by two levels if very serious limitations were found.

### Statistical analysis

The intervention effect was estimated using the dichotomized measure of risk ratio (RR) to evaluate the effect of clinical governance models. Where possible, we adopted the adjusted RR and pooled adjusted estimates from the original studies. Estimates were summarized if at least three studies reported the association of interest.

Heterogeneity between study-specific estimates was tested using *X*^2^ statistics [19] and measured with the *I*^2^ index (a measure of the percentage variation across the studies) [20]. Meta-analyses were conducted to combine the outcome data using the DerSimonian random effects model [21], which takes into account both the sampling variance within the studies and the variation in the underlying effect across studies, such as sample characteristics. Furthermore, subgroup analyses according to RCTs were carried out. A publication bias was tested using Egger’s regression and funnel plot visual analysis [22].

All tests were considered statistically significant for p-values less than 0.05. The analyses and the correspondent graphical visualization of forest and funnel plots were respectively performed by using RevMan V.5.4 (Nordic Cochrane Center) and STATA Software Program V.16.1 (STATA).

## Results

### Study selection

**Fig. 1:**
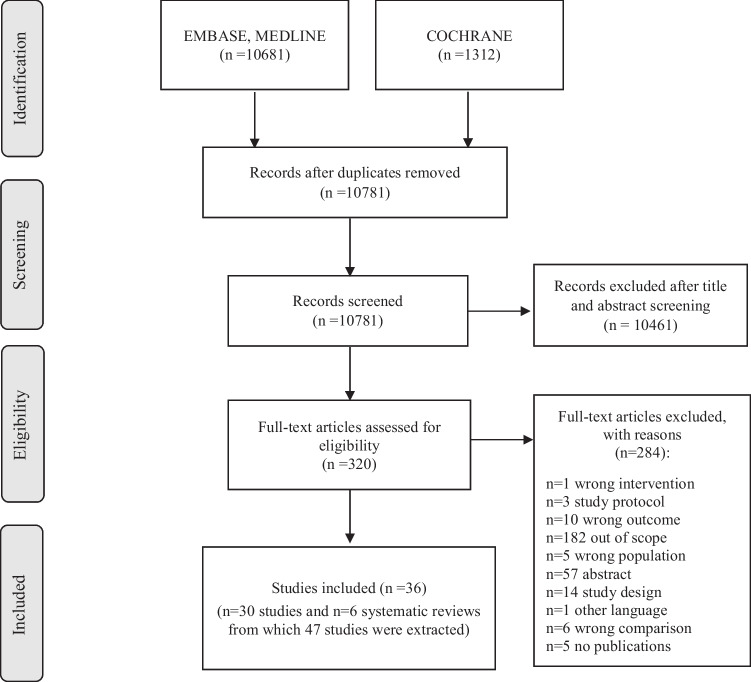
Flowchart of study selection

The objective of this study was to evaluate the efficacy of clinical governance implementation. A systematic literature review was carried out using the Embase, Medline, and Cochrane Central databases to update the clinical question elaborated by the SIGN Guideline [13]. As shown in Fig. 1, we identified 10,781 records.

We excluded 10,461 studies because they were unrelated to the issue based on the title and/or abstract. Among the remaining 320 publications assessed for full-text review, we excluded the studies that (i) considered the wrong population (5), intervention (1), comparison (5), or outcome (10); (ii) were study protocol (3) or abstract (57); (iii) had a wrong study design such as letter or case report (14); (iv) were out of scope (182) or not published in the English language (1). Further, the full text of five studies was not available. The remaining 36 publications were considered for the analysis, respectively: 30 primary studies [23–52] and 6 systematic reviews [53–58], from which 47 studies [59–105] were extracted (Table 1; Supplemental Material, Table S3).

**Table 1:** Characteristics of included studies

Author	Study type	Intervention/*N*	Control/*N*	Follow-up period (months)
Specialized model vs comparator model				
Coventry 2017	A retrospective analysis	Geriatric or comanaged model	Orthopedic model	12
Majumdar 2011	RCT	Patients followed by a nurse case-manager, who contacted patients and made clinic appointments for in-person visits and undertook several activities.	Patients followed by a multifaceted intervention, where patients received brief telephone-based counseling, and primary care physicians were faxed patient-specific reminders that notified them that their patient had been treated for a fragility fracture and that this put them at risk of osteoporosis	6
Vaculík 2017	Cohort study with survey	Patients with individual recommendations given to the patients and their GPs (detailed recommendation group)	Patients without individual recommendations (general recommendation group)	6
van Helden 2007	A prospective observational study	Patients admitted to a reference hospital in which a specialist osteoporosis nurse is employed	Patients admitted to five surrounding hospitals without a nurse	11-16 weeks
Heilmann 2012	Retrospective, parallel-group, cohort study	At the intervention site, a decentralized clinical-pharmacy-based osteoporosis management service (CPOMS) intervened on postmenopausal women following fracture	One centrally located registered nurse reviewed medical records for all women in the comparison group each month, assessed the appropriateness of either BMD screening or initiation of osteoporosis therapy, and sent recommendations to each patient’s primary care provider via the EMR	6
Kamel 2000	A retrospective chart review	Patients were seen by a medical consultant	Patients were not seen by a medical consultant	24
Rolnick 2001	RCT	Group 1 education class on osteoporosis Group 2 education plus BMD	No intervention	22
Sietsema 2018	A retrospective cohort study	Patients who received osteoporosis management service (OP MS) care with a follow-up visit within 90 days of the first fracture	Patients who did not seek OP MS care but had a physician visit within 90 days of the first fracture	12
Streetan 2006	A retrospective review of the charts	Patients received a consultation	Patients did not receive a consultation	48
Brankin 2005	Observational study	Those who had sustained a fracture or had ≥ 2 osteoporosis risk factors and had not previously been screened for osteoporosis were invited for a dual-energy X-ray absorptiometry scan.	A second group of participants were women within the Coatbridge Local Health Community Cooperative, who had been referred for a DXA scan by their GP on the basis of having risk factors for osteoporosis	18
Jachna 2003	A retrospective chart review	Hospitalists consultation at discharge	No consultation	na
FLS vs comparator model				
Murray 2005	Cohort study	Patients admitted at a centre with a formal fracture liaison service (FLS) responsible for screening fracture patients for osteoporosis	Patients admitted at other centre relied upon individual clinicians to initiate investigation or treatment for osteoporosis in patients following fracture	6
Ganda 2014	RCT	6-monthy follow-up in secondary fracture prevention (SFP) program (FLS).	Patients referral to their primary care physician with a single SFP program visit at 24 months	24
Wallace 2011	Practice in two fracture units was audited and compared using the NICE guidelines as an audit standard	Site B is a tertiary referral trauma centre which utilizes a continuous acute orthogeriatric care model for ward patients (one associate specialist 5 days per week, and one staff grade 3.5 days per week). In addition there are three consultant geriatrician-led ward rounds per week	In Site A, care is provided by trauma department doctors and a staff-grade orthogeriatrician (3.5 days per week) who will provide a medical assessment of a significant proportion of the unit’s inpatients. Consultant geriatrician ward-level assessment is available on a referral basis	na
Aubry-Rozier 2018	A nationwide survey	Patients followed by a FLS team	Patients followed by GP	12
Majumdar 2018	A pragmatic patient-level parallel-arm comparative effectiveness trial	High intensity FLS with a nurse-led case manager	Low intensity FLS with a low intensity multi-faceted intervention	6
Before-after specialized model				
Jones 2005	A retrospective audit	Patients followed after the introduction of the protocol	Patients followed before the introduction of the protocol	24
Laslett 2007	Retrospective study	Patients admitted after the implementation of a clinical pathway	Patients admitted before the implementation of a clinical pathway	12
Hawker 2003	Pre-post intervention study	Patients admitted to a simple fracture clinic intervention	Controls patients were selected from among fracture clinic attendees in the 6–9 months preceding the intervention	Intervention: 3 Control: 3–9
Huntjens 2011	Before–after impact analysis	Intervention group enrolled in 2004–2006 where a dedicated fracture nurse systematically offered fracture risk evaluation and treatment according to available guidelines	Pre-intervention group enrolled in 1999–2001	24
Schuijt 2020	A retrospective cohort study	Patients admitted to the orthogeriatric trauma unit, implemented on the first of January 2018	Historical cohort before the implementation of the orthogeriatric trauma unit	12
Astrand 2012	Cohort study with survey	Patients admitted from 2002 in a screening program at orthopedics department, where they are assessed by DEXA of the hip and spine, encouraged to see their doctor for decision on treatment regarding osteoporosis, and received written documents containing information, DEXA results, and a letter to their doctor with suggestions regarding blood tests and treatment	A historical control group of patients presented at department 1 year before the screening intervention	72
Anderson 2017	Pre–post study design	Patients admitted at a comprehensive geriatric hip fracture program	Patients admitted before the program implementation	na
Anighoro 2020	A retrospective cohort study	Patients admitted after the implementation of a standardized multidisciplinary pathway	Patients admitted before the implementation of a standardized multidisciplinary pathway	1
Lamb 2017	A retrospective review of a single institution’s outcomes	Injured patients admitted after the fragility fracture program implementation in 2015.	Patients admitted from 2014 who presented before implementation of the fragility fracture program	na
Roy 2011	Cohort study	Patients admitted after the implementation of an integrated model of care	Patients admitted before the implementation of an integrated model of care	na
Fisher 2006	Prospective observational study with a retrospective (historical) control.	In 1998, a geriatric medicine registrar began overseeing daily medical care with weekly geriatrician consultant review (prospective study)	Between 1995 and 1997, medical problems were managed by a geriatric medicine consultation-only service (retrospective audit)	48
Sofie 2016	Retrospective, single-centre study	Patients admitted to the orthopaedic ward after (October through December 2013) implementation of the clinical pathway	Patients admitted to the orthopaedic ward before (October through December 2010) implementation of the clinical pathway	na
Soong 2016	A retrospective pre–post study	The post-intervention period was from January 1, 2012 to December 31, 2013. This group receiving an integrated medical-surgical co-management incorporating continuous improvement methodology	The pre-intervention period was from January 1, 2009 to December 31, 2010	24
Sidwell 2004	An audit	Patients admitted to the orthogetriatric rehabilitation ward after the new protocol implementation	Comparisons were made with a similar group from the same service and same ward but prior to implementation of the protocol	na
Hofflich 2014	Pre-post observational study	Patients followed by a multidisciplinary team (September 1, 2009–June 30, 2010)	Patients followed in the pre-intervention period (July 1, 2008–June 30, 2009)	12
Johnson 2005	Prospective study	A simple intervention in a general orthopedic clinic	6-month pre-intervention group (October 2001 to March 2002)	6
Brañas 2018	Observational study	In 2012, a process management systems (PMS) was adopted to improve the quality of care, compliance, and efficiency, implementing it in January 2013	Patients admitted during the pre-process period (January 1, 2009, to December 31, 2012) orthogeriatric co-management model	inhospital deaths
Beaton 2017 “Improvements in osteoporosis..”	An interrupted time series analysis	The intervention consisted of assigning a screening coordinator to selected fracture clinics to identify, educate, and follow-up with fragility fracture patients and inform their physicians of the need to evaluate bone health.	At the control hospitals, no specific additions were made to the fracture management program and any care was dependent on the initiative of the usual health care team and their practices.	12
Ruggiero 2015	A prospective observational study	Patients treated by a Fracture Prevention Service (FPS), a multidisciplinary integrated model of care	Patients admitted in the pre-intervention phase	12
Tosi 2008	A pre-intervention and post-intervention study	Patients admitted to the Own the Bone project	Historical data collection	10
Jaglal 2009	Historical control, non-equivalent, pre-post intervention study	Patients admitted at the Integrated post-fracture care model (1 January to 31 December 2005)	Historical controls who received usual care during the year preceding the intervention (1 January to 31 December 2003)	Mean: Intervention: 17 Control: 8
Abrahamsen 2019	A prospective observational cohort study with historical control	Patients admitted to a orthogeriatric unit with interprofessional team consisting of orthopaedic surgeons, geriatric specialists, nurses, nursing assistants, physiotherapists, occupational therapists, and dieticians	Historical cohort (September 1, 2013 to January 31, 2014)	1
Baroni 2019	A single-center, pre-post intervention observational study	The intervention consisted of implementation of an orthogeriatric comanagement (OGC) and a geriatric consultation service (GCS) that took place from September 1st, 2011, to February 28th, 2012	The traditional orthopedic control group was obtained from the database of hospital records by looking at patients consecutively admitted to the same ward from March 1st to August 31st, 2011	12
Svenoy 2020	A single-center cohort study with historical controls	The patients in the hip fracture unit (HFU) group were included from May 2014 to May 2015	The patients in the control group were included from September 2009 to January 2012 These patients were the group randomized to “usual care”, i.e., admission to the orthopedic ward.	12
Before-after FLS				
Davidson 2017	Prospective cohort study with an historical control.	Patients admitted during the first 12 months after FLS implementation	Patients admitted during the 4 months before FLS implementation	36
Singh 2019	A controlled before-and-after study	At the end of February 2015, the FLS program was implemented, and the intervention group was recruited from the time of FLS implementation to February 2016 (approximately 12 months of recruitment). The intervention group received the FLS program integrated into their orthopaedic clinic visit	Participants were recruited into the study before the FLS program was implemented (approximately 5 months of recruitment; October 2014 to February 2015) and they formed the control group (receiving ‘usual care’)	6
Bachour 2017	A retrospective comparative study	Patients admitted after FLS implementation	Patients admitted before FLS implementation	24
Amphansap 2020	A prospective cohort study	Patients participated in FLS program from April 1, 2014–March 31, 2019 (5 years implementation).	The data were compared with a previous study, before the commencement of the FLS	12
Hawley 2016	Population-based longitudinal study with before–after time-series design	Orthogeriatric and nurse-led FLS models	Patients admitted before models implementation	24
Cosman 2017	Cohort study	Patients admitted after the FLS implementation, between February 2010–May 2011	The pre-FLS cohort included patients admitted for rehabilitation between July 2009 and February 2010	6
Wasfie 2019	A retrospective chart review with a pre-post study design	Patients who presented between January 2015 and December 2017, after fracture liaison service (FLS)	Patients who presented between January 2012 and December 2014, before FLS	24
Rotman-Pikielny 2018	A prospective study with historical controls	All patients hospitalized with hip fractures from February to August 2013 when a collaborative Orthopedic-Metabolic team was established, without a coordinator.	The historical controls included hip fracture patients hospitalized from February to August 2012	12
Axelsson 2016	Cohort study	Patients followed during 2013–2014 by FLS	Historic counterparts in 2011–2012 at the same hospital	24
Amphansap 2016	A prospective cohort study	Patients admitted after the FLS implementation from April 1, 2014-March 30, 2015. at the Police General Hospital, Bangkok	Patients admitted from a previous study prior to commencement of the FLS project	12
Chan 2015	‘Before and after’ cross-sectional extractions	Patients followed 12 months after 01/04/2009, the date that the primary care fracture liaison nurse started	Patients followed 12 months before 01/04/2009	12
Greenspan 2018	Pre–post study design	The FLS comparison included a prospective study of patients identified with an acute low-trauma fracture followed over six months for the same outcomes assessed above, but with the aid of the FLS model of care and the cloud-based tool. Patients were enrolled between April and December 2014	The baseline assessment included a retrospective chart review to obtain data on the number of adults who received bone mineral density studies, vitamin D testing, calcium/vitamin D supplementation, and appropriate osteoporosis therapy within six months following a recently diagnosed acute low-trauma fracture	6
Beaupre 2020	A population-based time series analysis	The H-FLS consisted of a nurse and physician team working where the nurse identified the patient for inclusion in the H-FLS and discussed the program with the patient and their family/caregiver as appropriate. The H-FLS programs were only offered to patients who resided in the local health zone pre-fracture	Patients admitted prior to H-FLS implementation to represent “usual care”	12
Specialized model vs standard care				
Beaton 2017	Cohort study	Cases came from the BMD fast track program that included full fracture risk assessment and communication of relevant guidelines to the primary care provider (PCP)	Controls were selected from the usual care program	6
Lih 2011	Prospective controlled observational study	Patients attending the minimal trauma fracture (MTF) program, a coordinated intervention program	Standard primary care	48
Cranney 2008	Cluster RCT	The effect of a multifaceted intervention, directed at both patient and primary care physician, was evaluated.	Usual care	6
Davis 2007	RCT	Patient Empowerment and Physician Alerting (PEPA) intervention	Usual care	6
Leslie 2012	RCT	Group 1 had mailed notification of the fracture sent to their primary care physicians and Group 2 had notifications sent to both physicians and patients	Usual care	12
Majumdar 2004	Non-randomized, controlled trial with blinded ascertainment of outcomes	Faxed physician reminders that contained osteoporosis treatment guidelines endorsed by local opinion leaders and patient education	Control patients received usual care and information about falls and home safety	6
Majumdar 2008	RCT	A multifaceted intervention directed at patients in the form of telephone-based education, and their physicians in the form of guidelines endorsed by opinion leaders, supported by reminders	Usual care	6
Morrish 2009	RCT	Case manager intervention	Usual care	12
Roux 2013	RCT	Group 1: minimal (MIN) intervention; Group2: intensive (INT) intervention.	Standard care	12
Yuksel 2010	RCT	Intervention consisted of printed materials, education, and quantitative ultrasound	Usual care	4
Merle 2017	RCT	Patients admitted at the post-fracture ‘Prevention of Osteoporosis’ (PREVOST) program, where trained case manager, repeated oral/written education, prompting to visit PCP were implemented	Usual care	6
Shigemoto 2018	A retrospective cohort study	Patients treated with a new multidisciplinary approach in 2014-2016	Patients received conventional treatment in 2012	12
Vidan 2005	RCT	Participants assigned to a daily multidisciplinary geriatric intervention	Usual care	12
Queally 2013	RCT	Patient admitted to fracture clinic	Usual care: assessment initiation by the participant’s general practitioner	3
Miki 2008	RCT	Inpatient osteoporosis evaluation initiated by orthopaedic surgeons combined with follow-up in a specialized orthopaedic osteoporosis clinic	Usual care where the responsibility of patients evaluation and treatment was placed solely on the primary care physician	6
Harrington 2005	Populatioon-based study	Direct referral pilot study (2002) where a nurse managed the direct referral process and contacted the patients to arrange DXA and osteoporosis consultation	Osteoporosis care by primary physicians (2000–2001)	12
FLS vs standard care				
Inderjeeth 2018	A prospective parallel cohort study	Patients admitted to FLS program	Routine care: retrospective group of the same hospital, and prospective group of other hospital	3-12
Naranjo 2017	Observational study	Orthogeriatric fracture liaison service (FLS)	Standard care	6
Huntjens 2014	Prospective study	FLS group	Non FLS group (standard fracture care)	24
Henderson 2017	Cohort study	Orthogeriatric service, a comprehensive geriatric assessment, daily medical involvement of a geriatric team and specialized follow-up assessment of bone and vascular health.	The comparative group received the usual standard of care, which consisted of standard orthopedic care with medical, or geriatric consults received on an as requested basis.	12
Specialized model vs non-attenders				
Van der Kallen 2014	Prospective cohort study	Patients who attended a Fracture Prevention Clinic	Patients who did not attend the clinic	24
Goltz 2013	Population-based study	Participants at program of integrated care for osteoporosis in terms of medication supply, fracture incidence and expenses	Controls were also diagnosed with osteoporosis but did not participate in the program	36
FLS vs non-attenders				
Nakayama 2016	Historical cohort study	FLS hospital	No FLS hospital	36
Sanli 2019	A prospective cohort-study	Attenders FLS	Non-attenders FLS	24

### Study characteristics

The majority of the studies were conducted in Australia (*n* = 10 [27, 36, 43, 59, 87–90, 94, 95]), Canada (*n* = 17 [25, 28, 34, 35, 38, 60–70, 102]), USA (*n* = 18 [23, 29, 31, 32, 37, 46, 77–86, 92, 93]), and European Union (*n* = 19 [24, 26, 33, 40, 42, 44, 45, 48–50, 76, 91, 96–98, 100, 101, 103, 105]). Five studies were carried out in the Asian continent (Israel, Japan, Thailand, and Lebanon), four publications were from the UK, three studies were performed in Ireland and one in New Zealand.

Fifteen studies were RCTs [28, 60–66, 72, 77, 92, 94, 100, 102, 105], while the remaining papers were observational studies.

Average follow-up was 9 and 17 months respectively for RCT and observational studies, although eight of them did not specify it [23, 24, 26, 32, 75, 83, 93, 104]. Studies were conducted using information from hospital or community-based hospitals or general practitioners [23–26, 28–34, 39–45, 48–50, 52, 59–61, 63, 66–71, 73, 75–79, 81–83, 86, 91, 93–95, 97–101, 103–105], tertiary referral hospital or centers [27, 36, 47, 88–90], or both [87], community pharmacies [64], administrative data [35, 37, 46, 51, 65, 70, 84, 85, 96] or specialized clinics or centers [62, 72, 74, 80, 92, 102]. In general, patients had low trauma fracture, specifically hip [23, 24, 27, 31, 32, 34, 35, 40–42, 44, 45, 47, 49, 51, 52], upper extremity [28], and vertebral [29] fracture.

Only one [46] of the observational studies extracted through the search had an NOS score lower than 6 and was therefore assigned to the category of low-quality study. Generally, “Comparability of cohorts on the basis of the design or analysis” in the comparability section was the domain for which problems were encountered in the most studies (14 [23, 24, 26, 30–32, 34, 35, 38, 39, 45–47, 52]), followed by the domain “Adequacy of follow-up of cohorts” in the outcome section (5 studies [25, 26, 34, 44, 46]) and “Demonstration that outcome of interest was not present at start of study” in the selection section (3 studies [25, 44, 46]).

Two systematic reviews [57, 58] were assessed as low quality, while the remaining were of very low quality (Supplemental Material, Table S4).

Studies considered the following comparisons: (a) after vs before the implementation of a specialized [23, 24, 26, 31, 32, 34, 38, 40, 44, 48, 49, 59, 67, 69, 76, 80, 82, 83, 85, 87, 88, 91, 97, 104] or a FLS [25, 29, 30, 35, 37, 39, 41, 43, 47, 51, 71, 86, 101] model, (b) a specialized model [27, 45, 46, 73, 78, 81, 84, 92, 93, 102, 103] or FLS [28, 33, 74, 75, 94] vs a comparator model, (c) a specialized [52, 60–66, 68, 70, 72, 77, 79, 89, 100, 105] or FLS [36, 42, 98, 99] model vs standard care or a specialized [90, 96] or FLS [50, 95] model vs non-attenders.

### Primary outcomes

**Fig. 2:**
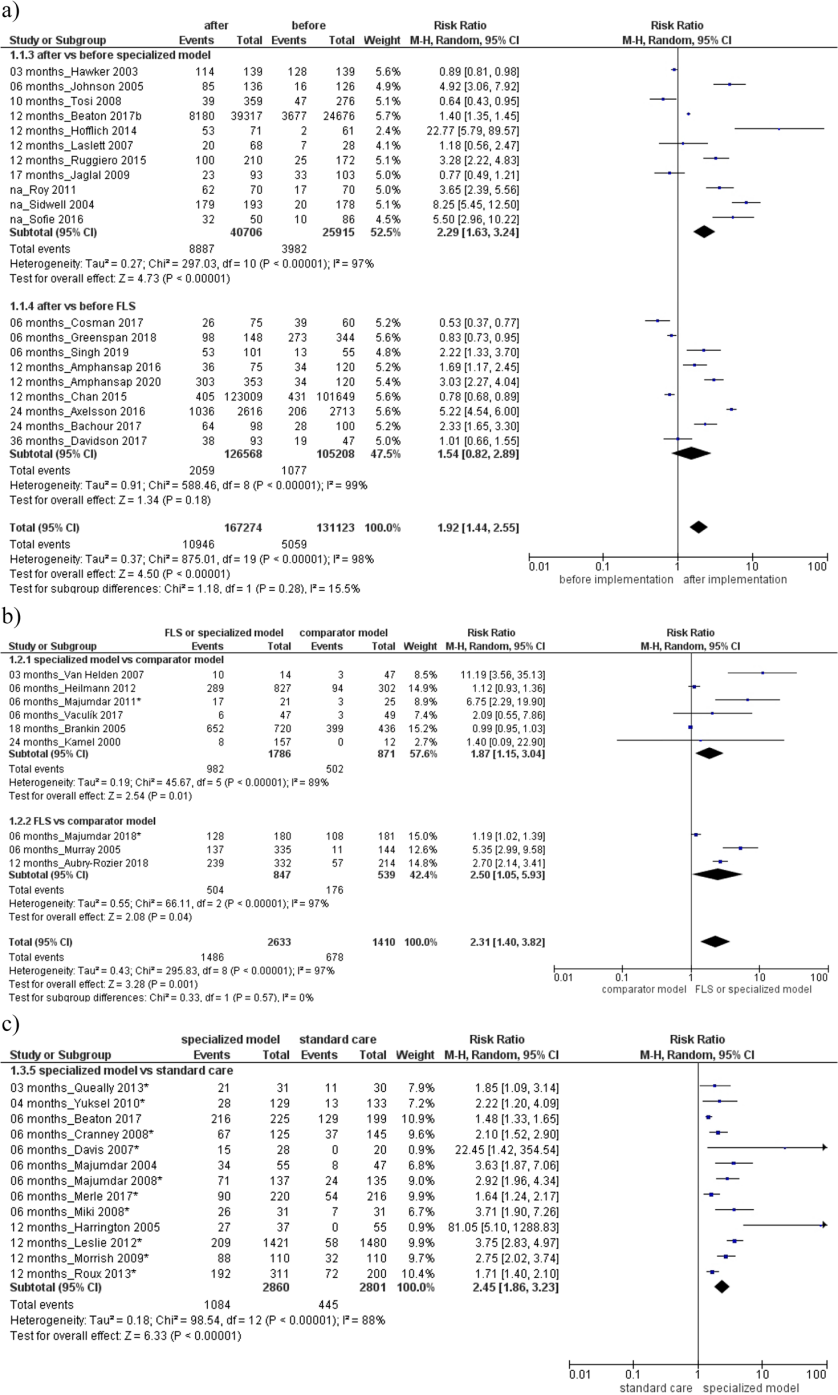
Evaluation of BMD testing rate **a** after vs before the specialized or fracture liaison service (FLS) model implementation, **b** in the specialized or FLS model vs comparator model, **c** in the specialized model vs standard care. Squares represent study-specific relative risk estimates (size of the square reflects the study-specific statistical weight, that is, the inverse of the variance); horizontal lines represent 95% CIs; diamonds represent summary relative risk estimates with corresponding 95% CIs; *p* values are from testing for heterogeneity between study-specific estimates. Asterisk indicates randomized controlled studies. Abbreviations: CI confidence interval, RR relative risk

As shown in Fig. 2a, increased BMD testing rate was detected after the implementation of a specialized model or FLS group compared their pre-implementation, respectively 10,946 and 5059. Overall, 20 studies detected a statistically significant RR of 1.92 (95% CI, 1.44 to 2.55) with a high heterogeneity between groups (*I*^2^ = 98%) and without publication bias (*p* = 0.29; Supplemental Material, Figure S1).

**Fig. 3:**
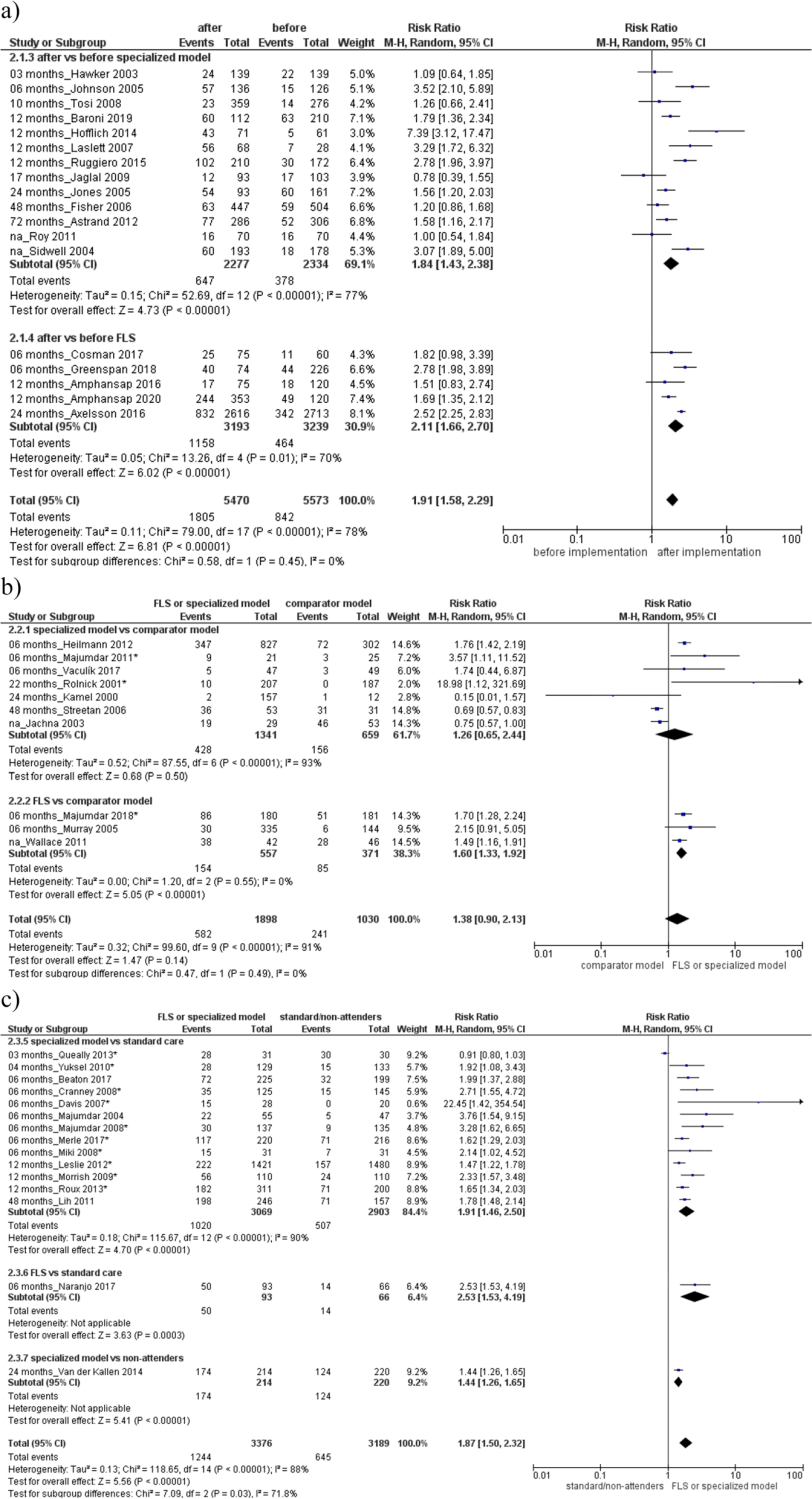
Evaluation of antiosteoporotic initiation **a** after vs before the specialized or fracture liaison service (FLS) model implementation, **b** in the specialized or FLS model vs comparator model, **c** in the specialized model or FLS vs standard care/non-attenders. Squares represent study-specific relative risk estimates (size of the square reflects the study-specific statistical weight, that is, the inverse of the variance); horizontal lines represent 95% CIs; diamonds represent summary relative risk estimates with corresponding 95% CIs; *p* values are from testing for heterogeneity between study-specific estimates. Asterisk indicates randomized controlled studies. Abbreviations: CI confidence interval, RR relative risk

**Fig. 4:**
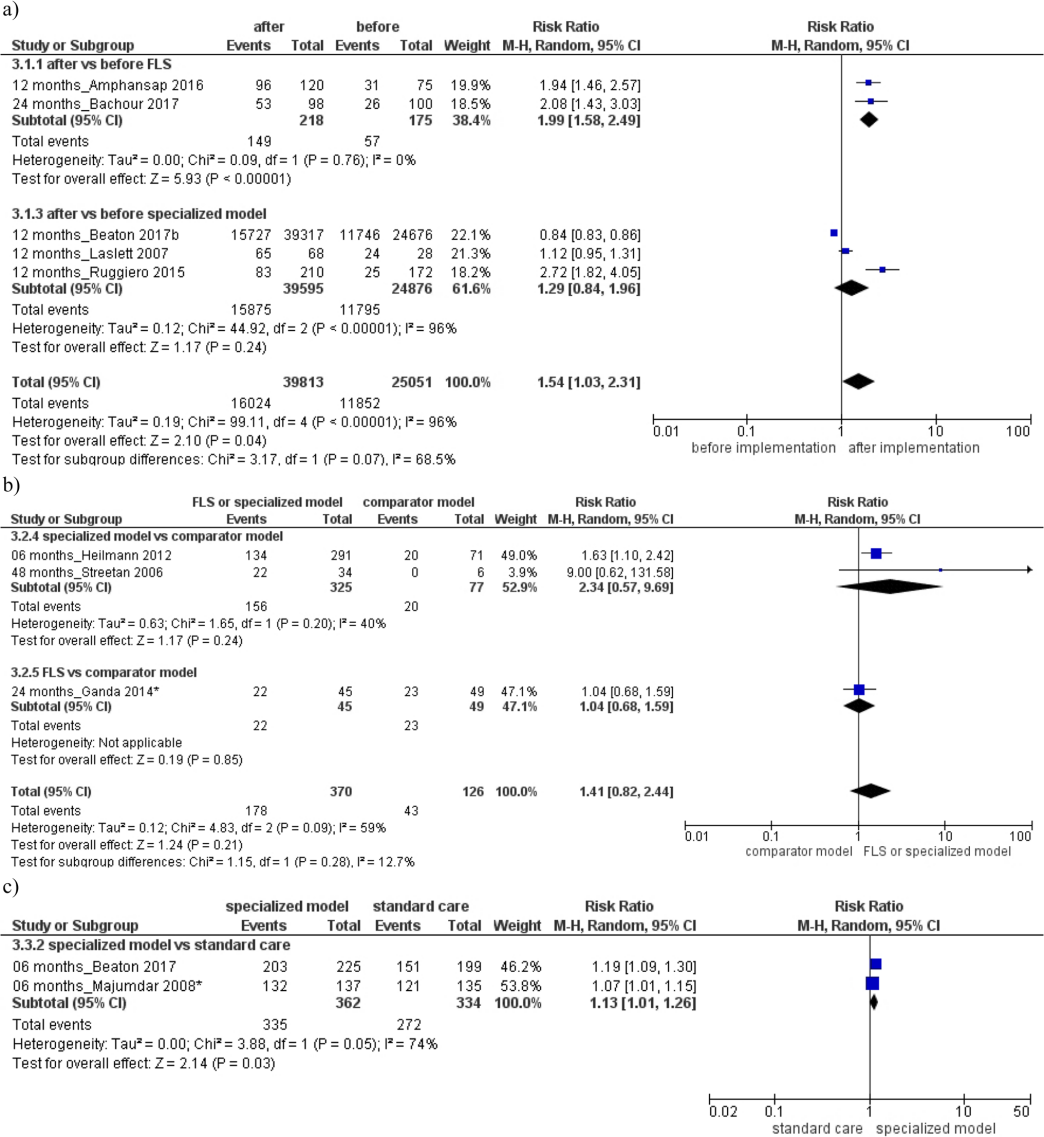
Evaluation of antiosteoporotic adherence **a** after vs before the specialized or fracture liaison service (FLS) model implementation, **b** in the specialized or FLS model vs comparator model, **c** in the specialized model vs standard care. Squares represent study-specific relative risk estimates (size of the square reflects the study-specific statistical weight, that is, the inverse of the variance); horizontal lines represent 95% CIs; diamonds represent summary relative risk estimates with corresponding 95% CIs; *p* values are from testing for heterogeneity between study-specific estimates. Asterisk indicates randomized controlled studies. Abbreviations: CI confidence interval, RR relative risk

Then, higher BMD testing rate was found in the specialized or FLS model respect to comparator model (Fig. 2b) RR 2.31 (95% CI, 1.40 to 3.82), or standard care (Fig. 2c) RR 2.45 (95% CI, 1.86 to 3.23), with a high heterogeneity among groups (*I*^2^ = 97% and 88%). Evaluation of antiosteoporotic therapy showed increased initiation after the specialized or FLS model implementation (RR 1.91, 95% CI 1.58 to 2.29; 18 studies, Fig. 3a) or compared to a standard care/non-attenders (RR 1.87, 95% CI 1.50 to 2.32; 15 studies, Fig. 3c). Furthermore, improved adherence to treatment was detected after the implementation of FLS or specialized model (RR 1.54, 95% CI 1.03–2.31; 5 studies, Fig. 4a) or compared to a standard care (RR 1.31, 95% CI 1.01 to 1.26; 2 studies, Fig. 4c). Both analyses were characterized by high heterogeneity among studies and absence of publication bias (Supplemental Material, Figure S1).

**Fig. 5:**
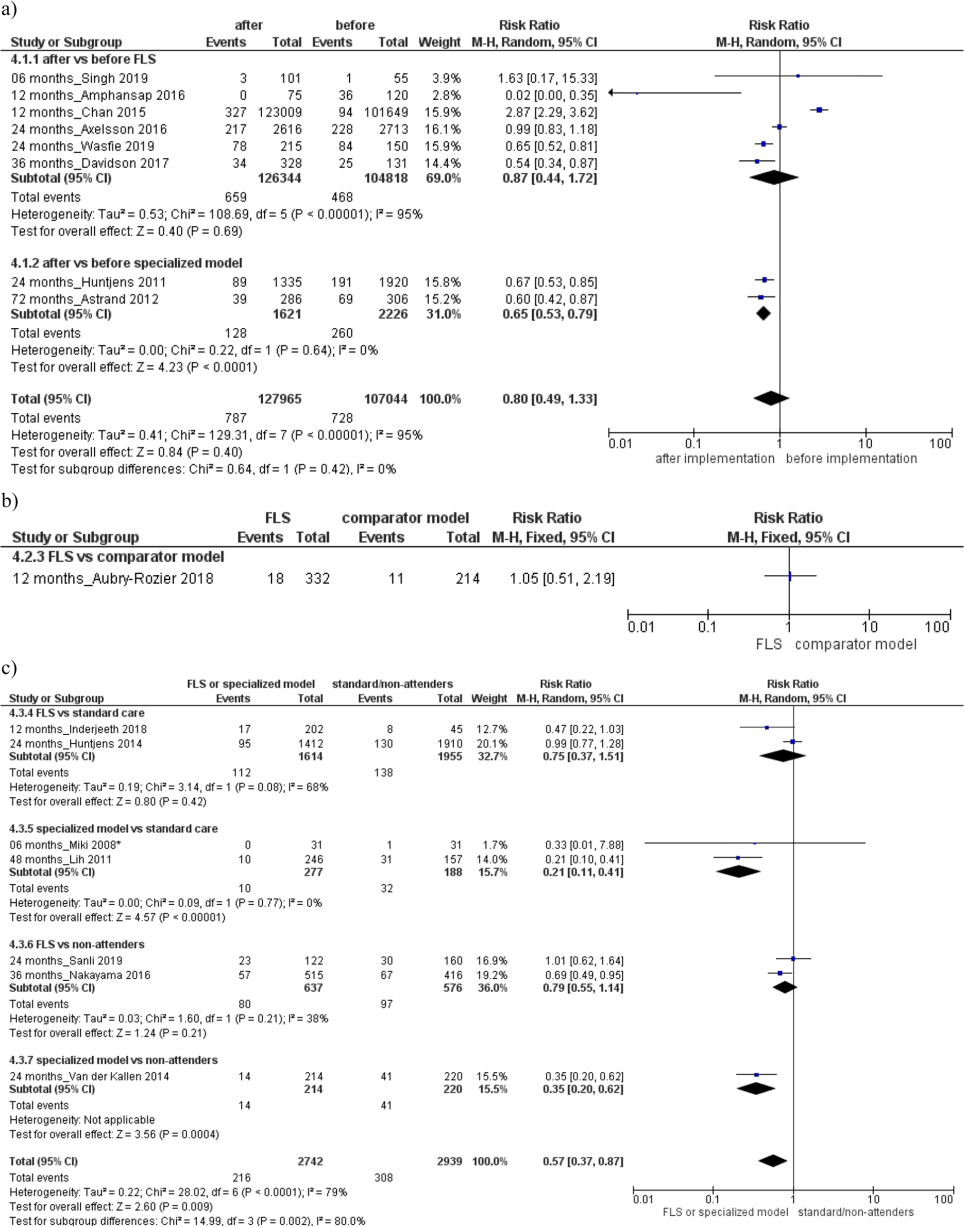
Evaluation of the risk of subsequent fracture **a** after vs before the specialized or fracture liaison service (FLS) model implementation, **b** in the FLS model vs comparator model, **c** in the specialized or FLS model vs standard care/non-attenders. Squares represent study-specific relative risk estimates (size of the square reflects the study-specific statistical weight, that is, the inverse of the variance); horizontal lines represent 95% CIs; diamonds represent summary relative risk estimates with corresponding 95% CIs; *p* values are from testing for heterogeneity between study-specific estimates. Asterisk indicates randomized controlled studies. Abbreviations: CI confidence interval, RR relative risk

**Fig. 6:**
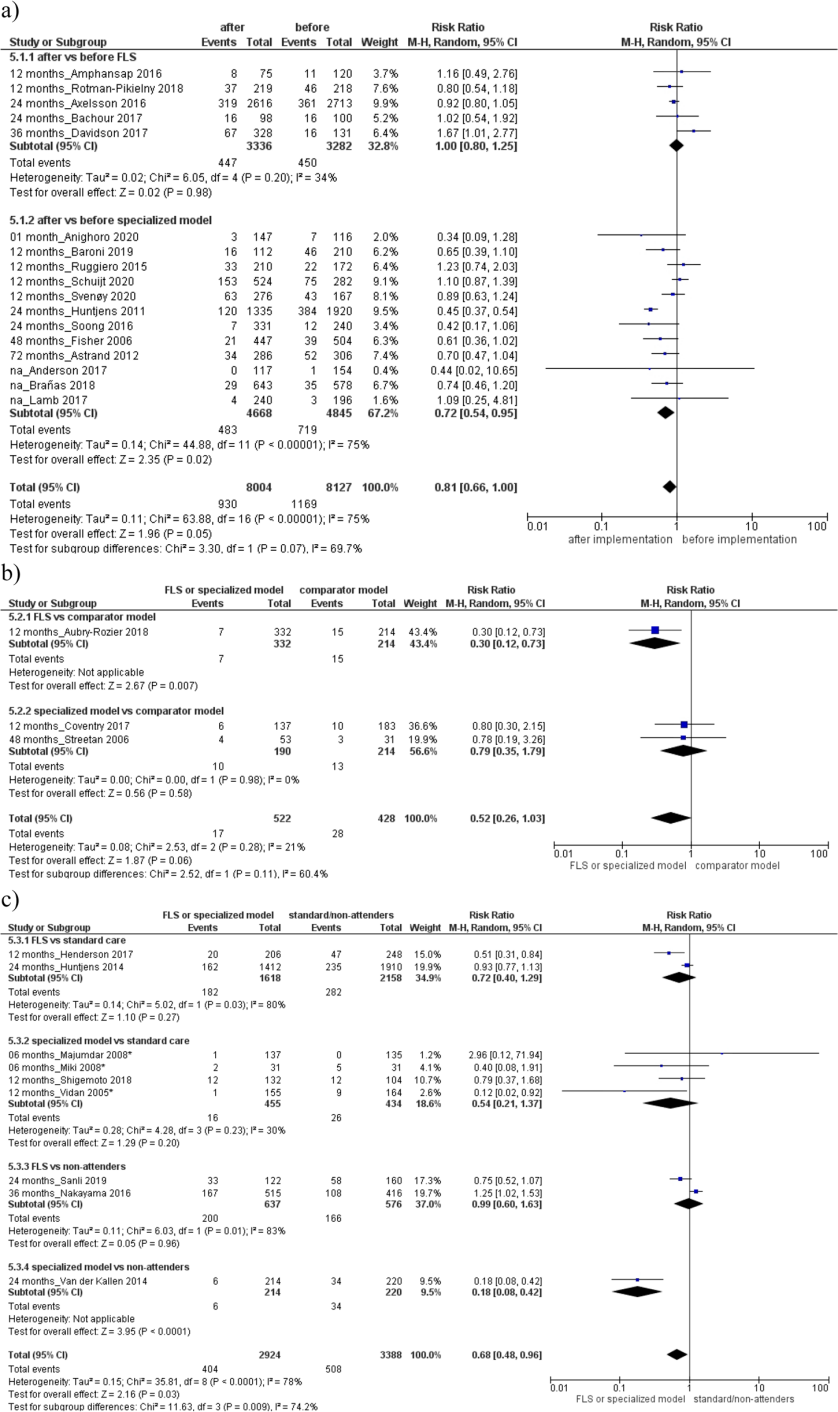
Evaluation of the risk of mortality **a** after vs before the specialized or fracture liaison service (FLS) model implementation, **b** in the specialized or FLS model vs comparator model, **c** in the specialized or FLS model vs standard care/non-attenders. Squares represent study-specific relative risk estimates (size of the square reflects the study-specific statistical weight, that is, the inverse of the variance); horizontal lines represent 95% CIs; diamonds represent summary relative risk estimates with corresponding 95% CIs; *p* values are from testing for heterogeneity between study-specific estimates. Asterisk indicates randomized controlled studies. Abbreviations: CI confidence interval, RR relative risk

Thus, a significant decreased risk of subsequent fracture and a reduction of the mortality rate was found after the specialized or FLS group implementation (subsequent fracture: RR 0.65, 95% CI 0.53 to 0.79; 2 studies; Fig. 5a; mortality: RR: 0.72, 95% CI 0.54 to 0.95; 12 studies, Fig. 6a) or respect to standard care/non-attenders (subsequent fracture: RR 0.57, 95% CI 0.37 to 0.87; 7 studies; Fig. 5c; mortality: RR 0.68, 95% CI 0.48-0.96; 9 studies; Fig. 6c). Both analyses were characterized by high heterogeneity between studies and no existence of publication bias (Supplemental Material, Figure S1).

For all of the aforementioned outcomes, the certainty of the evidence was downgraded from low to very low due to serious inconsistency and study design (Supplemental Material, Table S5).

All the above mentioned results are summarized in Supplemental Material, Table S6.

### Subgroup analyses

Previous findings regarding the BMD testing rate and antiosteoporotic initiation were confirmed based only on RCTs, specifically for the specialized or FLS model implementation compared to standard care/non-attenders (Supplemental Material, Figures S2-3). Moreover, an increased antiosteoporotic initiation was found for the specialized or FLS model implementation respect to a comparator model (Supplemental Material, Figure S3). Conversely, the summary estimate of the RCTs showed a non-significant reduction in the adherence to antiosteoporotic treatment, risk of subsequent fracture or mortality (Supplemental Material, Figure S2-6).

### Secondary outcomes

A systematic review [58] evaluated the effect of clinical care pathways that enrolled patients of over 50 years of age who had sustained a hip fracture. Twenty-two studies evaluated these secondary preventive measures compared to usual care. Twelve studies measured the health-related quality of life (HRQoL) between 3 and 12 months, which improved compared with usual care patients following hip fracture. Moreover, 19 studies estimated the physical function between 3 and 12 months that increased with respect to standard treatment. When the meta-analyses were stratified by length of follow-up, a greater HRQoL measure and physical function were found compared to usual care between 3 and 12 months.

## Discussion

This systematic review evaluated a clinical question of the Italian Guideline [106] and a panel of experts formulated recommendations through a structured and transparent process. Specifically, we conducted a systematic review and meta-analysis on the efficacy of clinical governance models (i.e., FLS, structured service delivery models, nurse-led clinics) versus the pre-implementation, a comparator model or standard care/non-attenders in low-income and developed countries. These results highlighted that implementation of clinical governance significantly improved BMD testing rate, antiosteoporotic therapy initiation, adherence as well as reduced the risk of subsequent fracture or mortality compared to the standard care/non-attenders. Moreover, a higher BMD testing rate, number of patients who initiated antiosteoporotic therapy and adherence to the medications was found after the FLS or specialized model implementation respect to their pre-implementation.

The benefits of the abovementioned results were more evident considering the RCT that underlined the effectiveness of the integrated structure of care versus standard treatment, specifically for the BMD testing rate and the antiosteoporotic initiation, or versus a comparator model, specifically for the antiosteoporotic initiation. These findings are consistent with studies that evaluated the implementation of an FLS, which similarly to our study underlined the effectiveness in reducing the bone fragility evaluation and treatment gaps, and subsequent fractures and mortality rates [57, 58, 107]. The results of this meta-analysis enabled us to recommend the management of patients with fragility fractures through multidisciplinary care systems (e.g., FLS) which ensures patients' transition to out-hospital services.

The primary objective of an FLS is the prevention of subsequent fragility fractures, associated with indirect and direct costs attributable to the antiosteoporotic treatment, which should be administered for prolonged periods to maintain therapy in subjects at high risk of fracture [5, 6]. Recently, the scientific community has focused on the impact of fragility fractures and their clinical consequences. Structures such as the multidisciplinary FLS are becoming increasingly popular in medical communities around the world. In the last decade, these programs have been promoted and supported by international scientific organizations, such as the International Osteoporosis Foundation (IOF), the American Society for Bone and Mineral Research (ASBMR) and the European League Against Rheumatism (EULAR) together with the European Federation of National Associations of Orthopaedics and Traumatology (EFORT) [9, 108–111]. International scientific societies have largely endorsed and promoted the establishment of coordinated, multidisciplinary clinical care governance for the management of patients with recent major fragility fractures in various parts of the world [112–116].

Regarding secondary outcomes, the establishment of clinical care pathways compared to usual care was demonstrated to improve HRQoL and physical performance in a meta-analysis that included patients over 50 years. This acquires particular importance in older patients with comorbidities and potentially improves the cost-effectiveness of these systems in clinical practice, given the various comorbidities displayed by these subjects.

### Limitations and strengths

Some limitations must be acknowledged. First, we considered different models of clinical governance, which may reduce the reliability of our findings. Moreover, the majority of studies were conducted in Europe or America, which may limit the generalizability of the results. Second, we have some concerns regarding heterogeneous multidisciplinary programs, characteristics of patients, fracture site at baseline, and length of follow-up. Third, the certainty of the evidence for the assessed outcomes was judged as “very low” or “low” due to the inconsistency of the estimates and the inclusion of observational studies with a modest sample size. Fourth, the majority of the included studies did not account for competing risks of death, which could have affected the results. Fifth, although falls may influence and increase the risk of subsequent fracture, this determinant was not an outcome of interest of the present meta-analysis. However, the role of falls will be investigated in a clinical question of the Italian Guideline and will be converted into a scientific article.

Despite the above limitations, this study presents points of strength. The exhaustive search strategy identified an overview of studies on the implementation of clinical governance programs. Then, the internal validity of the included studies was assessed using the Newcastle-Ottawa Scale for observational studies and the RoB tool for RCTs. Finally, preliminary performance indicators of FLS efficacy might be represented by BMD testing rate and initiation of treatment [109].

### Perspectives

Rigorous RCT testing the efficacy and effectiveness of models of clinical governance in secondary fracture prevention (i.e., FLS) against “standard care” will not likely be furtherly pursued in the future, mainly for ethical reasons. Therefore, longitudinal, large “real-world” studies, preferably designed and homogenized for including specific Key Performance Indicators of the efficacy of FLS, as advised by the international initiative IOF Capture The Fracture initiative-Best Practice Framework [117], are expected to be included in future systematic analyses in this field to reinforce the results. With this respect, also results coming from the surveys carried out within National Registries, which are now at an advanced stage of development worldwide [118–123], will be likely incorporated in these future assessments.

## Conclusion

This systematic review and meta-analysis indicate that the implementation of structured and integrated models of care increased the BMD testing rate, antiosteoporotic initiation and adherence to medication as well as reduced the risk of subsequent fracture and mortality and improved HRQoL and the physical function of patients experiencing a fragility fracture. The task force formulated recommendations on the introduction of these programs, although our systematic review judged outcomes affected by “very low” to “low” quality evidence.

### Supplementary information

ESM 1 Supplemental Table S1. Prisma Checklist. (PDF 888 kb)

ESM 2 Supplemental Table S2. Search Strategy. Supplemental Table S3. Characteristics of included studies. Supplemental Table S4. Quality evaluation. Supplemental Table S5. Summary of findings, GRADE approach. Supplemental Table S6. Summary results. Supplemental Figure S1. Funnel plot and Egger’s test. Supplemental Figure S2. BMD testing rate in FLS, RCT studies. Supplemental Figure S3. Antiosteoporotic initiation in FLS, RCT studies. Supplemental Figure S4. Antiosteoporotic adherence in FLS, RCT studies. Supplemental Figure S5. Subsequent fracture risk in FLS, RCT studies. Supplemental Figure S6. Mortality risk in FLS, RCT studies. Complete list of experts involved. (DOCX 353 kb)
