# Supplementary material for: The integrated structure of care: evidence for the efficacy of models of clinical governance in the prevention of fragility fractures after recent sentinel fracture after the age of 50 years
Source: Arch Osteoporos. 2023 Aug 21;18(1):109. doi: 10.1007/s11657-023-01316-9 (PMC10442313; doi:10.1007/s11657-023-01316-9)
Supplement: Supplementary file 2 — Supplemental Table S2. Search Strategy. Supplemental Table S3. Characteristics of included studies. Supplemental Table S4. Quality evaluation. Supplemental Table S5. Summary of findings, GRADE approach. Supplemental Table S6. Summary results. Supplemental Figure S1. Funnel plot and Egger’s test. Supplemental Figure S2. BMD testing rate in FLS, RCT studies. Supplemental Figure S3. Antiosteoporotic initiation in FLS, RCT studies. Supplemental Figure S4. Antiosteoporotic adherence in FLS, RCT studies. Supplemental Figure S5. Subsequent fracture risk in FLS, RCT studies. Supplemental Figure S6. Mortality risk in FLS, RCT studies. Complete list of experts involved. (DOCX 353 kb) [file 11657_2023_1316_MOESM2_ESM.docx]

**Supplemental Material**

Sommario

[Supplemental Table S2 1](#_Toc135998004)

[Search strategy 1](#_Toc135998005)

[Supplemental Tables S3 9](#_Toc135998006)

[Characteristics of included studies 9](#_Toc135998007)

[Supplemental Tables S4 49](#_Toc135998008)

[Quality evaluation 49](#_Toc135998009)

[Supplemental Tables S5 53](#_Toc135998010)

[Summary of Findings, GRADE approach 53](#_Toc135998011)

[Supplemental Tables S6 64](#_Toc135998012)

[Summary Results 64](#_Toc135998013)

[Supplemental Figure S1 66](#_Toc135998014)

[Funnel plot and Egger’s test 66](#_Toc135998015)

[Supplemental Figure S2 69](#_Toc135998016)

[BMD testing rate, RCT studies 69](#_Toc135998017)

[Supplemental Figure S3 70](#_Toc135998018)

[Antiosteoporotic initiation, RCT studies 70](#_Toc135998019)

[Supplemental Figure S4 71](#_Toc135998020)

[Antiosteoporotic adherence, RCT studies 71](#_Toc135998021)

[Supplemental Figure S5 71](#_Toc135998022)

[Refracture risk, RCT studies 71](#_Toc135998023)

[Supplemental Figure S6 72](#_Toc135998024)

[Mortality risk, RCT studies 72](#_Toc135998025)

[Complete list of experts involved 73](#_Toc135998026)

# Supplemental Table S2

## Search strategy

**MEDLINE SEARCH**:

#1:

(((wrist* or colles or radius or articulatio radiocarpea or carpus or carpal or radiocarp* or radial or forearm* or humerus or metacarp* or barton or monteggi* or ulna or ulnar or upper limb* or hip or hips or trochanteric or intertrochanteric or subtrochanteric or femoral neck or femur neck or spine or spinal or vertebra or vertebral or vertebrae or lumbar or shoulder* or glenohumeral or humeroscapular or scapulo humeral or proximal humeral) adj3 fractur*) or (exp hip fractures/ or spinal fractures/ or shoulder fractures/ or osteoporotic fractures/ or exp radius fractures/) or (fractures, bone/ and (exp wrist joint/ or exp spine/ or shoulder/ or shoulder joint/ or hip/))) and (exp osteoporosis/ or (osteoporo* or bone loss*))

#2:

“fragility fracture”[ti] OR “fragility fractures”[ti] OR “low energy fracture”[ti] OR “low energy fractures”[ti] OR “low-energy fracture”[ti] OR “low-energy fractures”[ti] OR “low trauma fracture”[ti] OR “low trauma fractures”[ti] OR “low-trauma fracture”[ti] OR “low-trauma fractures”[ti] OR “low energy trauma”[ti] OR “low-energy trauma”[ti] OR “low level trauma”[ti] OR “low-level trauma”[ti] OR “minor trauma fracture”[ti] OR “minor trauma fractures”[ti] OR “minor-trauma fracture”[ti] OR “minor-trauma fractures”[ti] OR “minor fracture”[ti] OR “minor fractures”[ti] OR “minor-fracture”[ti] OR “minor-fractures”[ti] OR “osteoporotic fracture”[ti] OR “osteoporotic fractures”[ti]

#3:

#1 OR #2

#4

(exp Patient Care Team/) AND fracture*[tiab] AND (fragil*[tiab] OR osteopor*[tiab)

#5

exp Preventive Health Services/ AND fracture*[tiab] AND (fragil*[tiab] OR osteopor*[tiab)

#6

((service*[Tiab] or program*[Tiab] or care[Tiab] or model*[Tiab] or intervention*[Tiab] or pathway*[Tiab]) AND (multifaceted[Tiab] or integrated[Tiab] or multimodal[Tiab] or multifaceted[Tiab] or coordinated[Tiab] or co-ordinated[Tiab])) AND (fracture*[tiab]) AND (fragil*[tiab] OR osteopor*[tiab)

#7

(care pathway*[Tiab] or treatment pathway*[Tiab] or management pathway*[Tiab]) AND fracture*[tiab] AND (fragil*[tiab] OR osteopor*[tiab)

#8

((service*[Tiab] or program*[Tiab] or care[Tiab]) AND delivery[Tiab]) AND (fracture*[tiab]) AND (fragil*[tiab] OR osteopor*[tiab)

#9

(nurse*[Tiab] AND (clinic[Tiab] or clinics[Tiab])) AND fracture*[tiab] AND (fragil*[tiab] OR osteopor*[tiab)

#10

(healthcare[Tiab] AND (delivery[Tiab] or model*[Tiab] or integrate*[Tiab])) AND (fracture*[tiab]) AND (fragil*[tiab] OR osteopor*[tiab)

#11

(health care[Tiab] AND (delivery[Tiab] or model*[Tiab] or integrate*[Tiab])) AND (fracture*[tiab]) AND (fragil*[tiab] OR osteopor*[tiab)

#12

(health service*[Tiab] AND (delivery[Tiab] or model*[Tiab] or integrate*[Tiab])) AND (fracture*[tiab]) AND (fragil*[tiab] OR osteopor*[tiab)

#13

(Recurrence/pc ) AND (fragil*[tiab] OR osteopor*[tiab) AND fracture*[tiab]

#14

((secondary fracture*[Tiab] or recurrent fracture*[Tiab] or subsequent fracture*[Tiab]) AND prevent*[Tiab]) AND (fragil*[tiab] OR osteopor*[tiab) AND fracture*[tiab]

#15

fracture*[Tiab] AND (clinic[Tiab] or clinics[Tiab]) AND (fragil*[tiab] OR osteopor*[tiab)

#16

fracture*[Tiab] AND (service*[Tiab] or team*[Tiab]) AND (fragil*[tiab] OR osteopor*[tiab)

#17

fracture liaison[Tiab] AND (fragil*[tiab] OR osteopor*[tiab) AND fracture*[tiab]

#18

after[Tiab] AND fracture*[Tiab] AND (fragil*[tiab] OR osteopor*[tiab])

#19

post[Tiab] AND fracture*[Tiab] AND (fragil*[tiab] OR osteopor*[tiab])

#20

postfracture[Tiab] AND (fragil*[tiab] OR osteopor*[tiab) AND fracture*[tiab]

#21

(pathway*[Tiab] or service*[Tiab] or program*[Tiab] or model*[Tiab]) AND (fracture*[tiab]) AND (fragil*[tiab] OR osteopor*[tiab)

#22

(discharge*[Tiab] AND (treat*[Tiab] or assess*[Tiab] or follow*[Tiab] or identif*[Tiab])) AND (fracture*[tiab]) AND (fragil*[tiab] OR osteopor*[tiab)

#23

#4 OR #5 OR #6 OR #7 OR #8 OR #9 OR #10 OR #11 OR #12 OR #13 OR #14 OR #15 OR #16 OR #17 OR #18 OR #19 OR #20 OR #21 OR #22

#24

(#3 AND #23) AND limit: Humans

**EMBASE search:**

#1:

'wrist fracture'/exp OR 'hip fracture'/exp OR 'spine fracture'/exp OR 'shoulder fracture'/exp OR 'fragility fracture'/exp OR 'radius fracture'/exp OR ((wrist* OR colle* OR radius OR 'articulatio radiocarpea' OR carpus OR carpal OR radiocarp* OR radial OR forearm* OR humerus OR metacarp* OR barton OR monteggi* OR ulna OR ulnar OR 'upper limb' OR 'upper limbs' OR hip OR hips OR trochanteric OR intertrochanteric OR subtrochanteric OR 'femoral neck' OR 'femur neck' OR spine OR spinal OR vertebra* OR lumbar OR shoulder* OR glenohumeral OR humeroscapular OR 'scapulo humeral' OR 'proximal humeral') NEAR/3 fractur*):ab,ti OR ('fracture'/exp AND ('wrist'/exp OR 'hip'/exp OR 'spine'/exp OR 'shoulder'/exp OR 'wrist injury'/de OR 'shoulder injury'/exp OR 'hip injury'/exp OR 'spine injury'/exp)) AND ('osteoporosis'/exp OR osteoporo*:ab,ti OR 'bone loss':ab,ti)

#2:

'fragility fracture'/exp

#3:

'low energy fracture'/exp

#4:

'low trauma fracture'/exp

#5:

'low energy trauma'/exp

#6:

“fragility fracture”:ti OR “fragility fractures”:ti OR “low energy fracture”:ti OR “low energy fractures”:ti OR “low-energy fracture”:ti OR “low-energy fractures”:ti OR “low trauma fracture”:ti OR “low trauma fractures”:ti OR “low-trauma fracture”:ti OR “low-trauma fractures”:ti OR “low energy trauma”:ti OR “low-energy trauma”:ti OR “low level trauma”:ti OR “low-level trauma”:ti OR “minor trauma fracture”:ti OR “minor trauma fractures”:ti OR “minor-trauma fracture”:ti OR “minor-trauma fractures”:ti OR “minor fracture”:ti OR “minor fractures”:ti OR “minor-fracture”:ti OR “minor-fractures”:ti OR “osteoporotic fracture”:ti OR “osteoporotic fractures”:ti

#7:

#1 OR #2 OR #3 OR #4 OR #5 OR #6

#8

(‘patient Care’) AND fracture*:ti,ab AND (fragil*:ti,ab OR osteopor*:ti,ab)

#9

(‘preventive Health Service’) AND fracture*:ti,ab AND (fragil*:ti,ab OR osteopor*:ti,ab)

#10

((service*:ti,ab or program*:ti,ab or care:ti,ab or model*:ti,ab or intervention*:ti,ab or pathway*:ti,ab) AND (multifaceted:ti,ab or integrated:ti,ab or multimodal:ti,ab or multifaceted:ti,ab or coordinated:ti,ab or co-ordinated:ti,ab)) AND (fracture*:ti,ab) AND (fragil*:ti,ab OR osteopor*:ti,ab)

#11

(care pathway*:ti,ab or treatment pathway*:ti,ab or management pathway*:ti,ab) AND fracture*:ti,ab AND (fragil*:ti,ab OR osteopor*:ti,ab)

#12

((service*:ti,ab or program*:ti,ab or care:ti,ab) AND delivery:ti,ab ) AND (fracture*:ti,ab) AND (fragil*:ti,ab OR osteopor*:ti,ab)

#13

(nurse*:ti,ab AND (clinic:ti,ab or clinics:ti,ab)) AND fracture*:ti,ab AND (fragil*:ti,ab OR osteopor*:ti,ab)

#14

(Healthcare:ti,ab AND (delivery:ti,ab or model*:ti,ab or integrate*:ti,ab)) AND (fracture*:ti,ab) AND (fragil*:ti,ab OR osteopor*:ti,ab)

#15

(health care:ti,ab AND (delivery:ti,ab or model*:ti,ab or integrate*:ti,ab)) AND (fracture*:ti,ab) AND (fragil*:ti,ab OR osteopor*:ti,ab)

#16

(health service*:ti,ab AND (delivery:ti,ab or model*:ti,ab or integrate*:ti,ab)) AND (fracture*:ti,ab) AND (fragil*:ti,ab OR osteopor*:ti,ab)

#17

(‘recurrence risk’) AND (fracture*:ti,ab) AND (fragil*:ti,ab OR osteopor*:ti,ab)

#18

((secondary fracture*:ti,ab or recurrent fracture*:ti,ab or subsequent fracture*:ti,ab) AND prevent*:ti,ab) AND fracture*:ti,ab AND (fragil*:ti,ab OR osteopor*:ti,ab)

#19

fracture*:ti,ab AND (clinic:ti,ab or clinics:ti,ab) AND (fragil*:ti,ab OR osteopor*:ti,ab)

#20

fracture*:ti,ab AND (service*:ti,ab or team*:ti,ab) AND (fragil*:ti,ab OR osteopor*:ti,ab)

#21

fracture liaison:ti,ab AND (fragil*:ti,ab OR osteopor*:ti,ab) AND fracture*:ti,ab

#22

after:ti,ab AND fracture*:ti,ab AND (fragil*:ti,ab OR osteopor*:ti,ab)

#23

post:ti,ab AND fracture*:ti,ab AND (fragil*:ti,ab OR osteopor*:ti,ab)

#24

postfracture:ti,ab AND (fragil*:ti,ab OR osteopor*:ti,ab) AND fracture*:ti,ab

#25

(pathway*:ti,ab or service*:ti,ab or program*:ti,ab or model*:ti,ab) AND (fracture*:ti,ab) AND (fragil*:ti,ab OR osteopor*:ti,ab)

#26

(discharge*:ti,ab AND (treat*:ti,ab or assess*:ti,ab or follow*:ti,ab or identif*:ti,ab)) AND (fracture*:ti,ab) AND (fragil*:ti,ab OR osteopor*:ti,ab)

#27

#8 OR #9 OR #10 OR #11 OR #12 OR #13 OR #14 OR #15 OR #16 OR #17 OR #18 OR #19 OR #20 OR #21 OR #22 OR #23 OR #24 OR #25 OR #26

#28

(#7 AND #27) AND limit: Humans

**COCHRANE SEARCH:**

1:

((wrist* or colle* or radius or "articulatio radiocarpea" or carpus or carpal or radiocarp* or radial or forearm* or humerus or metacarp* or barton or monteggi* or ulna or ulnar or "upper limb" or "upper limbs" or hip or hips or trochanteric or intertrochanteric or subtrochanteric or "femoral neck" or "femur neck" or spine or spinal or vertebra* or lumbar or shoulder* or glenohumeral or humeroscapular or "scapulo humeral" or "proximal humeral") near/3 fractur*):ti,ab or [mh "hip fractures"] or [mh "spinal fractures"] or [mh "shoulder fractures"] or [mh "osteoporotic fractures"] or [mh "radius fractures"] or ([mh "bone fractures"] and ([mh "wrist joint"] or [mh spine] or [mh shoulder] or [mh "shoulder joint"] or [mh hip])) and ([mh osteoporosis] or (osteoporo* or "bone loss" OR fragility):ti,ab)

#2:

MeSH descriptor: [Osteoporotic Fractures] explode all trees

#3:

MeSH descriptor: [Fractures, Spontaneous] explode all trees

#4:

(fragility fracture):ti OR (fragility fractures):ti OR (low energy fracture):ti OR (low energy fractures):ti OR (low-energy fracture):ti OR (low-energy fractures):ti OR (low trauma fracture):ti OR (low trauma fractures):ti OR (low-trauma fracture):ti OR (low-trauma fractures):ti OR (low energy trauma):ti OR (low-energy trauma):ti OR (low level trauma):ti OR (low-level trauma):ti OR (minor trauma fracture):ti OR (minor trauma fractures):ti OR (minor-trauma fracture):ti OR (minor-trauma fractures):ti OR (minor fracture):ti OR (minor fractures):ti OR (minor-fracture):ti OR (minor-fractures):ti OR (osteoporotic fracture):ti OR (osteoporotic fractures):ti OR (pathologic fracture):ti OR (pathological fractures):ti

#5:

#1 OR #2 OR #3 OR #4

#6

“patient Care”:ti,ab OR “patient-care”:ti,ab OR fracture liaison:ti,ab OR clinic:ti,ab OR clinics:ti,ab OR service*:ti,ab OR team*:ti,ab OR care pathway*:ti,ab OR treatment pathway*:ti,ab OR management pathway*:ti,ab OR after:ti,ab OR post:ti,ab OR postfracture:ti,ab pathway*:ti,ab OR service*:ti,ab OR program*:ti,ab OR model*:ti,ab

#7

#6 AND (fracture*:ti,ab AND (fragil*:ti,ab OR osteopor*:ti,ab))

#8

#5 AND #7

#9

#8 with Cochrane Library publication date from Jan 2013 to present

#10

#9 NOT ((MH "Animals+") OR (MH "Animal Studies") OR (TI "animal model*"))

# Supplemental Tables S3

## Characteristics of included studies

| **Study** | **Bell 2014 Arch Osteoporos** |
| --- | --- |
| Study type | Systematic review |
| Number of studies/ number of participants | 3 trials and 15 observational studies |
| Settings | Cohort studies, cohort pre/post, prospective observation study, RCT cluster randomized trial, RCT, and observational studies.  Studies conducted on humans in any outpatient environment. |
| Funding | The salary for Ms. Kate Bell has been partially funded by an unrestricted grant from Novartis Pharmaceuticals |
| Duration of study | Search up to April 2013 |
| Age, gender, ethnicity | The included studies were conducted on adult patients aged from 40 to 100 years. |
| Patient characteristics | Adult patients (aged ≥ 40 years) who have low trauma fracture; |
| Intervention | Any outpatient environment (fracture clinic, emergency department) |
| Outcomes | - Effect of a dedicated osteoporosis health professional on screening and treatment |

| **Study** | **Chang 2018 Osteoporosis International** |
| --- | --- |
| Study type | Systematic review |
| Number of studies/ number of participants | 37 studies |
| Settings | Randomized controlled trials, observational studies with control groups, pre-post, cross-sectional. Studies conducted on humans |
| Funding | This work was supported by Amgen Inc. |
| Duration of study | Search up to February 2017 |
| Age, gender, ethnicity | The included studies were conducted on adult patients aged 55 years and older from Asia-Pacific regions. |
| Patient characteristics | Adult patients (aged ≥ 55 years) with all types of osteoporosis-related fractures; |
| Intervention | Fracture liaison services |
| Outcomes | - To identify the treatment gaps in current fracture liaison services - To provide recommendations for best practice establishment of future FLS across the Asia-Pacific region |

| **Study** | **Ganda 2013 Osteoporos Int** |
| --- | --- |
| Study type | Systematic review |
| Number of studies/ number of participants | 10 randomised trials (including 1 cluster randomised controlled trial), 5 observational studies, 11 before and after studies, 1 cross-sectional analytical study |
| Settings | Randomized controlled trials, observational studies with control groups, pre-post, cross-sectional. Studies conducted on humans |
| Funding | This work was supported by Amgen Inc. |
| Duration of study | Search up to 2011 |
| Age, gender, ethnicity | The included studies were conducted on adult patients aged 45 years and older from Asia-Pacific regions. |
| Patient characteristics | Adult patients (aged ≥ 45 years) with all types of osteoporosis-related fractures; |
| Intervention | in-patients departments:   - orthopaedic wards - outpatient departments - orthopaedic clinics - emergency departments - a combination of the latter - radiology practices |
| Outcomes | - To improve the care of people who gave sustained minimal trauma fractures - To reduce the incidence of fracture fractures - To critically appraise the available studies on models of care in order to establish specific features associated with effective secondary fracture prevention programs |

| **Study** | **Wu 2018 Osteoporosis International** |
| --- | --- |
| Study type | Systematic review |
| Number of studies/ number of participants | 20 RCTs and 37 controlled observational studies |
| Settings | Randomized and non-randomized phase 1-4 trials, retrospective or prospective observational studies  Studies conducted on humans in FLS |
| Funding | This work was supported by Amgen Inc. |
| Duration of study | Search up to February 2017 |
| Age, gender, ethnicity | The included studies were conducted on adult patients aged 50 years and older. |
| Patient characteristics | Adult patients (aged ≥ 50 years) with osteoporosis-related fractures; |
| Intervention | Fracture liaison services |
| Outcomes | - To evaluate the evidence describing the structure and format of FLS interventions - To identify the characteristics that lead to optimal patient outcomes |

| **Study** | **Wu 2018 Bone** |
| --- | --- |
| Study type | Systematic review |
| Number of studies/ number of participants | 16 RCTs and 58 observational studies |
| Settings | Randomized and non-randomized phase 1-4 trials, retrospective or prospective observational studies.  Studies conducted on humans in FLS |
| Funding | This work was supported by Amgen Inc. |
| Duration of study | Search up to February 2017 |
| Age, gender, ethnicity | The included studies were conducted on adult patients aged 50 years and older. |
| Patient characteristics | Adult patients (aged ≥ 50 years) with all types of osteoporosis-related fractures; |
| Intervention | Fracture liaison services |
| Outcomes | - To evaluate the outcomes of patients with osteoporosis-related fractures managed through fracture liaison services (FLS) programs - To update, critically reevaluate, and quantify the available evidence on the incidence of BMD testing, treatment initiation, adherence, re-fractures, and rates of mortality associated with FLS in patients with osteoporosis |

| **Study** | **A multidisciplinary approach to improve the quality of care for patients with fragility fractures**  **Lamb 2017** |
| --- | --- |
| Study type | Retrospective review |
| Number of studies/ number of participants | N= 437 |
| Settings | Acute Inpatient Medical Service, Academic medical center, USA |
| Funding | None |
| Duration of study | Search up to December 2015 |
| Age, gender, ethnicity | Age (mean): not reported  Gender (% F): 2014 group: 68.4%, 2015 group: 68.8%  Ethnicity (% asian, % black, % hispanic, % white): 2014 group: 1%, 6.6%, 3%, 88.3%, 2015 group: 0.8%, 5%, 2.9%, 90% |
| Patient characteristics | Adult patients (aged ≥ 50 years) with an isolated hip fracture from a low velocity mechanism |
| Intervention | Patients were stratified into two groups: those from 2014 who presented before implementation of the fragility fracture program (N=) and those who were injured and admitted after the fragility fracture program was in place in 2015 (N=) |
| Outcomes | - to identify and implement best practices in order to reduce geriatric fragility fracture complications |

| **Study** | **Building for better bones: evaluation of a clinical pathway in the secondary prevention of osteoporotic fractures**  **Sofie 2016** |
| --- | --- |
| Study type | Retrospective, single-centre study |
| Number of studies/ number of participants | N = 172 |
| Settings | General hospital AZ Sint-Jan Brugge-Oostende AV, Belgium |
| Funding | None |
| Duration of study | Search up to February 2017 |
| Age, gender, ethnicity | Age (median): before implementation group:79, after implementation group:82  Gender (% F): before implementation group: 77%, after implementation group: 70%  Ethnicity: not reported |
| Patient characteristics | Adult patients (aged ≥ 50 years) admitted to the orthopaedic surgery unit of the general hospital with a low energy fracture |
| Intervention | Patients were were divided into two groups based on the period of admission to the hospital: before (N=86) and after (N=50) the implementation of a clinical pathway; |
| Outcomes | - To identify and to treat osteoporosis and consequently prevent secondary fractures |

| **Study** | **Comparison of 3 different perioperative care models for patients with hip fractures within I health service**  **Coventry 2017** |
| --- | --- |
| Study type | Retrospective study |
| Number of studies/ number of participants | N = 183 |
| Settings | Western Health, Australia |
| Funding | The author(s) received no financial support for the research, authorship, and/or publication of the article |
| Duration of study | Between November 2012 and March 2014 |
| Age, gender, ethnicity | Age (median): orthopedic model: 84, geriatric model: 83, comanaged model: 82  Gender (% F): orthopedic model: 69.4%, geriatrc model: 71.5%, comanaged model: 72.2%  Ethnicity: not reported |
| Patient characteristics | Adult patients (aged ≥ 65 years) with hip fracture treated at Western Health; |
| Intervention | Patients were admitted under the orthopedic model (N=183), under the geriatric model (N=137), and under the comanaged model (N=126) |
| Outcomes | - To evaluate differences in perioperative care between 3 models - To identify the association with length of stay and additional patient outcomes for the hip fracture patients |

| **Study** | **From ER to OR: results after implementation of multidisciplinary pathway for fragility hip fractures at a level I trauma center**  **Anighoro 2020** |
| --- | --- |
| Study type | Retrospective review |
| Number of studies/ number of participants | N = 263 |
| Settings | Level I trauma hospital |
| Funding | The author(s) received no financial support for the research, authorship, and/or publication of the article |
| Duration of study | Not reported |
| Age, gender, ethnicity | Age (mean): pre: 82, post: 83  Gender (% F): pre:63.8%, post:74.8%  Ethnicity: not reported |
| Patient characteristics | Adult patients (aged ≥ 65 years) diagnosed with a fragility hip fracture; patients were included if they had an isolated femoral neck, intertrochanteric, or subtrochanteric fracture sustained through a low-energy mechanism and/or his or her power of attorney desired surgical treatment;periprosthetic fractures, pathologic fractures, patients with high-energy mechanisms with associated acetabular fractures, and polytrauma patients were excluded |
| Intervention | Patients were divided accordingly to the time of admission to the hospital if it was before (N=116) or after (N=147) the implementation of a multidisciplinary hip fracture pathway |
| Outcomes | - To analyze patient outcomes after the implementation of a multidisciplinary hip fracture pathway at a level I trauma center |

| **Study** | **Geriatric hip fracture care: fixing a fragmented system**  **Anderson 2017** |
| --- | --- |
| Study type | Comprehensive geriatric hip fracture program |
| Number of studies/ number of participants | N = 172 |
| Settings | University of Colorado Hospital |
| Funding | None |
| Duration of study | Search up to October 2014 |
| Age, gender, ethnicity | Age(median): preintervention:80.9, postintervention:79.5  Gender(% F): preintervention: 73%, postintervention: 62%  Ethnicity: not reported |
| Patient characteristics | Adult patients (aged ≥ 65 years) who sustained an acute hip fracture after minimal trauma; they excluded patients with nonfragility hip fractures, nonhip femur fractures, periprosthetic fractures, or pathologic fractures |
| Intervention | Three interventions:   - admission of all ward-status patients with hip fractures to the single Orthopedic Surgery Service with hospitalist comanagement, including nonoperative cases - geographic placement of patients with hip fractures on the Orthopedic Unit - standardization of care   Patient were identified as before (pre intervention (N=154)) or after (post intervention (N=117)) implementation of the program |
| Outcomes | - To describe a stepwise approach to system redesign for this patient population |

| **Study** | **Impact of an integrated hip fracture inpatient program on length of stay and costs**  **Soong 2016** |
| --- | --- |
| Study type | Retrospective, single-centre pre-post study |
| Number of studies/ number of participants | N = 571 |
| Settings | Mount Sinai Hospital (MSH), Toronto, Canada |
| Funding | None |
| Duration of study | Between January 2009 and December 2013 |
| Age, gender, ethnicity | Age (mean): before group: 80.1, after group: 79.4  Gender (% F): before group: 69.2%, after group: 71.3%  Ethnicity: not reported |
| Patient characteristics | Adult patients (aged ≥ 18 years) with a primary diagnosis of hip fracture;patients with the diagnoses of pathological or periprosthetic fractures were excluded |
| Intervention | Patients entered in the before i-HIP group (N=240) if they were admitted to the hospital before the implementation of the program, otherwise they entered in the after i-HIP group (N=331) |
| Outcomes | - To determine whether an integrated interprofessional co-management care model of hip fracture patients would improve outcomes while reducing costs |

| **Study** | **Improvements in osteoporosis testing and care are found following the wide scale implementation of the Ontario Fracture Clinic Screening Program. An interrupted time series analysis**  **Beaton 2017** |
| --- | --- |
| Study type | Retrospective, single-centre pre-post study |
| Number of studies/ number of participants | N = 147071 |
| Settings | Ontario, Canada |
| Funding | None |
| Duration of study | Between January 2002 and March 2010 |
| Age, gender, ethnicity | Age(%50-65, %66-80, %80+): before: control:25.4%, 35.2%, 39.5%, intervention:27.7%, 35.2%, 37.1%, after: control: 27.1%, 32.1%, 400.7%, intervention: 30%, 32.3%, 37.6%  Gender (% F): before: control: 75.3%, intervention: 74.3%, after: control: 74.5%, intervention:74.5%  Ethnicity: not reported |
| Patient characteristics | Adult patients (aged ≥ 50 years) with low energy fractures; |
| Intervention | The intervention consisted of assigning a screening coordinator to selected fracture clinics to identify, edicate, and follow-up with fragility fracture patients and inform their physicians of the need to evaluate bone health. 37 hospitals were assigned a screening coordinator. 23 similar hospitals were control sites. Patients were divided into before (control hospitals N=24676, intervention hospitals N=69856) and after (control hospitals N=13222, intervention hospitals N=39317) groups |
| Outcomes | - to evaluate the impact of the implementation of the Fracture Clinic Screening Program of the Ontario Osteoporosis Strategy on BMD testing, medication initiation, and medication persistence in the year after a fragility fracture |

| **Study** | **Beyond orthogeriatric co-management model: benefits of implementing a process management system for hip fracture**  **Brañas 2018** |
| --- | --- |
| Study type | Trial |
| Number of studies/ number of participants | N = 1221 |
| Settings | Hospital Universitario Infanta Leonor, Spain |
| Funding | None |
| Duration of study | Between January 2009 and December 2016 |
| Age, gender, ethnicity | Age (mean): preprocess: 83.2, process: 84.6  Gender (% F): preprocess: 77.5%, process: 76.3%  Ethnicity: not reported |
| Patient characteristics | Adult patients (aged ≥ 65 years) admitted to the hospital for acute hip fracture surgery; |
| Intervention | According to the time of admission to the hospital, patients were divided into the preprocess group (N=578) or the process group (N=643) |
| Outcomes | - To assess the effectiveness of the PMS applied to hip fracture versus the orthogeriatric co management model in the acute phase |

| **Study** | **Comparing strategies targeting osteoporosis to prevent fractures after an upper extremity fracture (C_STOP trial): a randomized controlled trial**  **Majumdar 2018** | |
| --- | --- | --- |
| Study type | Patient-level parallel-arm comparative effectiveness trial | |
| Number of studies/ number of participants | N = 361 | |
| Settings | Canada | |
| Funding | This trial received funding from Alberta Innovates through a Partnership in Research and Innovation in the Healthcare System grant and in-kind support from the Alberta Strategy for Patient-Oriented Research Support Unit | |
| Duration of study | Between January 2002 and March 2010 | |
| Age, gender, ethnicity | Age (mean): active control: 63, case manager: 63  Gender (% F): active control: 90%, case manager: 88%  Ethnicity: not reported | |
| Patient characteristics | Community-dwelling patients (aged ≥ 50 years) with upper extremity fractures who were not on bisphosphonate treatment; patients who sustained a pathological or multiple fractures, lived outside of the metropolitan health zone at time of fracture, were unable to understand or converse in English, or were unable to provide written informed consent were excluded | |
| Intervention | Low intensity multi-faceted intervention was the active control (N=181), nurse-led case manager was the case manager (N=180) | |
| Outcomes | - To initiate bisphosphonate treatment within 6 month of fragility fracture - To assess whether a BMD test and a composite measure were completed within 6 months of a fracture - To assess health status and upper extremity specific functional outcomes as well as disease-specific HRQL - To estimate intervention costs | |
| **Study** | **Implementation of an in-patient hip fracture liaison services to improve initiation of osteoporosis medication use within 1-year of hip fracture: a population-based time series analysis using the RE-AIM framework**  **Beaupre 2020** |  |
| Study type | Population-based cohort study |  |
| Number of studies/ number of participants | N = 1427 |  |
| Settings | 2 hospitals in Alberta, Canada |  |
| Funding | This study was supported by an Alberta Innovates Partnership for Research and Innovation in the Health System grant , a Covenant Health Research Grant |  |
| Duration of study | Between January 2002 and March 2010 |  |
| Age, gender, ethnicity | Age (mean F): pre-implementation: 78.8, post-implementation: 80.6  Gender (% F): pre-implementation: 70%, post-implementation: 70.7%  Ethnicity: not reported |  |
| Patient characteristics | Adult patients (aged ≥ 50 years) that underwent hip fracture surgery at 1 or 2 tertiary hospitals in a Canadian province and survived to 12 months post-fracture; those with a post-admit diagnosis of hip fracture, procedural codes of revisions, procedures in centers other than surgical hospital, or whose fracture was managed non-operatively were exclude |  |
| Intervention | Patients were included in the pre-implementation group (N=583) or in the post-implementation group (N=597) |  |
| Outcomes | - to examine implementation of an in-patient hip fracture liaison service to improve osteoporosis medication use after hip fracture using the RE-AIM framework |  |
| **Study** | **Improving osteoporosis management in primary care: an audit of the impact of a community based fracture liaison nurse**  **Chan 2015** |  |
| Study type | audit |  |
| Number of studies/ number of participants | N = 18677 |  |
| Settings | 12 practices in the southeast of England |  |
| Funding | This audit was funded by Crawley Practice Based Commissioning (PBC) group |  |
| Duration of study | The study was conducted in 2010 |  |
| Age, gender, ethnicity | Age: 50 years and above  Gender (% F): 100%  Ethnicity: not reported |  |
| Patient characteristics | Adult women (aged ≥ 50 years) with a code diagnosis or an associated operation, or a computer record only used in osteoporosis |  |
| Intervention | Patients were divided into two groups: female 50-74 (N=14520), and females ≥75 years old (N=4157) |  |
| Outcomes | - to audit the impact of a primary care based fracture liaison nurse on the detection of fragility fractures in people with osteoporosis and their treatment with a bone-sparing medication |  |

| **Study** | **Orthopedic-metabolic collaborative management for osteoporotic hip fracture**  **Rotman-Pikielny 2018** |
| --- | --- |
| Study type | Prospective study |
| Number of studies/ number of participants | N = 219 |
| Settings | Israel |
| Funding | Merck Pharmaceuticals provided financial support for the statistical analysis |
| Duration of study | Between February 2012 and August 2013 |
| Age, gender, ethnicity | Age (mean): 2012 group: 82.2, 2013 group: 83.5  Gender (% F): 2012 group: 71.6%, 2013 group: 74.9%  Ethnicity: not reported |
| Patient characteristics | Adult patients (aged ≥ 50 years) with a code diagnosis or an associated operation, or a computer record only used in osteoporosis; patients were excluded if they were younger than 60 years, misdiagnosed, or had a secondary hip fracture during the study period or 2 hospitalizations for a single hip fracture |
| Intervention | Patients were divided into two groups according to the time of admission to the hospital: Feb-Aug 2012 (N=218), Feb-Aug 2013 (N=219) |
| Outcomes | - To attend the Metabolic Clinic - Osteoporosi diagnosis, vitamin D measurement and treatment, referral to the Metabolic Clinic - To initiate osteoporosis treatment during the first visit - 1-year mortality rate |

| **Study** | **Prevention of osteoporotic refractures in regional Australia**  **Davidson 2017** |
| --- | --- |
| Study type | Prospective cohort study with an historical control |
| Number of studies/ number of participants | N = 140 |
| Settings | Australia |
| Funding | None |
| Duration of study | Between september 2011 and September 2012 |
| Age, gender, ethnicity | Age (mean): cohort: 71.3, control: 75.8  Gender (% F): cohort: 75.3%, control: 80.9%  Ethnicity: not reported |
| Patient characteristics | Adult patients (aged ≥ 45 years) who were admitted with a MTF; patients were excluded if they had a pathological fracture or if they were deceased |
| Intervention | Control (N=47) and cohort (N=93) groups comprised patients consenting to interview who presented with a MTF to the major referral hospital 4 months before and 12 months after FLS implementation respectively |
| Outcomes | - To evaluate the effectiveness of the nurse-led FLS |

| **Study** | **Secondary fracture prevention in hip fracture patients requires cooperation from general practitioners**  **Vaculík 2017** |
| --- | --- |
| Study type | Observational cohort study |
| Number of studies/ number of participants | N = 207 |
| Settings | Orthopedic Department, Bulovka Hospital, Prague, Czech Republic |
| Funding | The study was supported by the Grant GZd CR 0002384101 |
| Duration of study | Between September 2010 and January 2011 |
| Age, gender, ethnicity | Age: not reported  Gender: not reported  Ethnicity: not reported |
| Patient characteristics | Adult patients (aged ≥ 50 years) who were hospitalized with a low-energy hip fracture; |
| Intervention | Two groups of patients: in the first one (N=111) general recommendations on osteoporosis treatment and fracture prevention were provided in a discharge report addressed to the GP, in the second one (N=96) patients were provided individually with a detailed written set of recommendations on osteoporosis examination, treatment, and fracture prevention, which was also provided in the discharge report |
| Outcomes | - To evaluate whether an individual recommendation on osteoporosis treatment addressed to a hip fracture patient’s GP would lead to better osteoporosis management |

| **Study** | **The effect of a multidisciplinary approach on geriatric hip fractures in Japan**  **Shigemoto 2018** |
| --- | --- |
| Study type | Report |
| Number of studies/ number of participants | N = 469 |
| Settings | Japan |
| Funding | None |
| Duration of study | Between 2014 and 2016 |
| Age, gender, ethnicity | Age (mean): conventional group: 84, multidisciplinary group: 84.6  Gender (% F): conventional group: 83%, multidisciplinary group: 81%  Ethnicity: not reported |
| Patient characteristics | Adult patients (aged ≥ 65 years) who presented at the hospital with a hip fracture ; |
| Intervention | Patients arrived at the hospital during 2 observational periods: conventional group (N=105) and multidisciplinary group (N=364) |
| Outcomes | - To report results of the multidisciplinary treatment approach for geriatric hip fractures - To evaluate its effectiveness compared with conventional treatment |

| **Study** | **The effectiveness of a private orthopaedic practice-based osteoporosis management service to reduce the risk of subsequent fractures**  **Sietsema 2018** |
| --- | --- |
| Study type | Retrospective cohort study |
| Number of studies/ number of participants | N = 1304 |
| Settings | U.S. Centers for Medicare & Medicaid Services (CMS), Michigan, USA |
| Funding | Eli Lilly and Company funded the study |
| Duration of study | Between April 2010 and September 2014 |
| Age, gender, ethnicity | Age(mean): exposed cohort: 75.4, unexposed cohort: 74.9  Gender (% F): exposed cohort: 68.9%, unexposed cohort: 71%  Ethnicity (% white, % balck): exposed cohort: 93.9%, 3.8%, unexposed cohort: 90.7%, 7.2% |
| Patient characteristics | Adult patients (aged ≥ 65 years) diagnosed with at least 1 medical claim including an ICD-9-CM, CPT, or HCPCS code for fractures; |
| Intervention | Patients with a follow-up OP MS visit with a participating orthopaedic physician or NP in Grand Rapids, Michigan, within 90 days following the fracture date were considered the exposed cohort (N=1306); patients from other areas of Michigan who did not receive OP MS care but who had follow-up physician visit within 90 days of the fracture date were considered the unexposed cohort (N=123815) |
| Outcomes | - To evaluate the effectiveness of a private orthopaedic practice-based osteoporosis management service (OP MS) in reducing subsequent fracture risk and improving other aspects of osteoporosis management of patients who had sustained fractures |

| **Study** | **The orthogeriatric comanagement improves clinical outcomes of hip fracture in older adults**  **Baroni 2019** |
| --- | --- |
| Study type | Pre-post observational study |
| Number of studies/ number of participants | N = 430 |
| Countries and settings | Santa Maria Misericordia hospital, Umbria, Italy |
| Funding | Not reported |
| Duration of study | Between September 2011 and February 2012 |
| Age, gender, ethnicity | Age(mean): OGC: 83.3, GCS: 82.4, UOC: 85.0  Gender (%F): OGC: 78.6%, GCS: 74.1%, UOC: 73.8%  Ethnicity: not reported |
| Patient characteristics | Adult patients aged 65 years or older, hospitalized because of a proximal native or low-impact femur fracture; patients with peri-prosthetic, cancer related, multiple trauma and inherited bone disorder fractures were excluded. |
| Intervention | Implementation of an OGC and a GCS. In the 6-months after the implementation of OGC and GCS models, data were prospectively gathered from participants consecutively admitted to the Trauma and Orthopedic Ward. As informed consent was obtained, participants were randomly assigned to OGC (N=112) or GCS (N=108) or UOC (n=210) by orthopedic resident on call, in collaboration with the orthopedic surgeon in charge, using the coin-flipping procedure |
| Outcomes | - To improve clinical outcomes among older people with hip fractures |

| **Study** | **The role of the Fracture Liaison Service (FLS) in subsequent fracture prevention in the extreme elderly**  **Sanli 2019** |
| --- | --- |
| Study type | Prospective cohort study |
| Number of studies/ number of participants | N = 282 (interest subgroup of patients with previous fractures N=103) |
| Countries and settings | The Netherlands |
| Funding | None |
| Duration of study | Between 2006 and 2011 |
| Age, gender, ethnicity | Age(mean): FLS attenders: 87, FLS non-attenders: 89  Gender (%F): FLS attenders: 84%, FLS non-attenders: 81%  Ethnicity: not reported |
| Patient characteristics | Adult patients aged 85 years or older with a clinical fracture, who were treated at the Maastricht University Hospital (European level-one trauma center) during a 5 year period; patients who died within 30 days were excluded, as were patients already treated for osteoporosis, patients with vertebral fractures, pathological fractures, as well as patients not currently living in the Netherlands or living in the Belgian boarder adjacent to Maastricht. |
| Intervention | In patients that attended the FLS, assessment of bone mineral density and fall-risk factors were screened. In both the attenders and non-attenders groups, mortality and subsequent fracture rates were scored during the follow-up |
| Outcomes | - To evaluate the subsequent fracture risk in all patient > 85 years, comparing the two populations of Fracture Liaison service (FLS) attended and non-attenders |

| **Study** | **Fracture liaison service: report on the first successful experience from the Middle East**  **Bachour 2017** |
| --- | --- |
| Study type | Retrospective comparative study |
| Number of studies/ number of participants | N = 250 |
| Countries and settings | Lebanon |
| Funding | Not reported |
| Duration of study | Between June 2014 and July 2016 |
| Age, gender, ethnicity | Age(mean): group A: 72.2, group B: 75.5  Gender (n. F): group A: 69, group B: 78  Ethnicity: not reported |
| Patient characteristics | Adult patients aged 50 years and above identified as having a minimal trauma fracture.There were no patients who had more than one incident fracture |
| Intervention | Patients are divided into two groups: group A (N=130) is composed of patients presenting before FLS implementation, and group B (N=120) is composed of patients presenting during the year following FLS implementation in the hospital |
| Outcomes | - To evaluate the effect of FLS implementation on bone health assessment, osteoporosis treatment maintenance, ad re-fracture rate reduction after an indexed minimal trauma fracture - To present an outcome-based proof in favor of diffusion of this model in Lebanese and Middle-eastern hospitals - The refracture rate in 2-year period follow-up following the indexed fracture - DEXA bone mineral density evaluation, osteoporosis treatment instauration, and death occurrence in this same period |
| **Study** | **Impact of a fracture liaison service on patient management after an osteoporotic fracture: the CHUV FLS**  **Aubry-Rozier 2018** |
| Study type | Osteocare study |
| Number of studies/ number of participants | N = 606 |
| Countries and settings | Eight hospitals in Switzerland |
| Funding | None |
| Duration of study | Between October 2008 and October 2011 |
| Age, gender, ethnicity | Age(mean): FLS group: 75.5, GP group: 79.5  Gender (%F): FLS group: 81%, GP group: 83%  Ethnicity: not reported |
| Patient characteristics | Adult patients who had been seen by the CHUV FLS for an osteoporotic fracture, who were alive at the time of hospital discharge and had provided written informed consent to participate in the study. Patients were excluded if they had severe dementia, an already known secondary cause of osteoporosis, refusal to participate, and follow-up by both their GP and FLS team |
| Intervention | One year after each patient’s acute fracture was registered by the FLS, data were collected on osteoporosis management and course/events. Patients followed up by their GP (N=274) were sent a written questionnaire, and the institutional database was used to collect information on all patients followed up by the FLS (N=332) |
| Outcomes | - To compare osteoporosis management of patients registered with the FLS between the two forms of management (FLS or GP) - To compare one- and five-year new fracture and mortality rates between patients managed by the FLS team and those managed by their GP |

| **Study** | **Implementation of the Western Australian Osteoporosis Model of Care: a fracture liaison service utilising emergency department information system to identify patients with fragility fracture to improve current practice and reduce re-fracture rates: a 12-month analysis**  **Inderjeeth 2018** |
| --- | --- |
| Study type | Prospective parallel cohort study |
| Number of studies/ number of participants | N = 1058 |
| Countries and settings | Australia |
| Funding | None |
| Duration of study | Between 2012 and 2014 |
| Age, gender, ethnicity | Age(mean): FLS: 71, PC: 71, RC: 70  Gender (%F): FLS: 81.7%, PC: 89.1%, RC: 72.4%  Ethnicity: not reported |
| Patient characteristics | Adult patients aged 50 years or older resident in WA who presented to the ED after suffering a MTF. Exclusion criteria included those whose fracture was not considered to be a MTF, patients in high-level residential aged care facilities, not permanent residents of WA, or those already entered in the retrospective arm of the study at SCGH. Patients with fractures of the hands, feet, or skull only were excluded |
| Intervention | Patients in the FLS (N=714) and prospective control cohort (N=137) were identified by the EDIS at baseline. Patients in the retrospective control group were identified through EDIS 3 months post discharge from ED (N=207) |
| Outcomes | - Self-reported MTF events following the indexed MTF - Self-reported patient awareness of osteoporosis and diagnosis, investigations performed, treatments started or modified, falls, health care utilisation, and quality of life |

| **Study** | **Implementing a fracture liaison service open model of care utilizing a cloud-based tool**  **Greenspan 2018** |
| --- | --- |
| Study type | Pre-post comparison of fracture care before and after implementation of the FLS program at three facilities |
| Number of studies/ number of participants | N = 492 |
| Countries and settings | USA |
| Funding | None |
| Duration of study | Between 2014 and 2015 |
| Age, gender, ethnicity | Age(mean): Site A: pre FLS N=70.8, post FLS N=68.6  Site B: pre FLS N= 68.9, post FLS N=67.4  Site C: pre FLS N= 71.9, post FLS N=70.9  Gender (n. F): Site A: pre FLS N=81, post FLS N=77  Site B: pre FLS N= 66, post FLS N=84  Site C: pre FLS N= 67, post FLS N=73  Ethnicity (% caucasian, %african american): Site A: pre FLS N=65%, 20%, post FLS N=62%, 10%  Site B: pre FLS N= 93%, 0%, post FLS N=95%, 5%  Site C: pre FLS N= 87%, 6%, post FLS N=69%, 7% |
| Patient characteristics | Adult patients aged 50 years or older with a recently diagnosed fracture; patients were excluded if they had sustained a traumatic fracture, had a cancer-related fracture, or were currently on osteoporosis therapy |
| Intervention | N=344 patients were included in the retrospective analysis; N=148 patients were included in the post  Site A: pre FLS N=99, post FLS N=60  Site B: pre FLS N= 100, post FLS N=43  Site C: pre FLS N= 145, post FLS N=45 |
| Outcomes | - To determine if secondary prevention of a recently diagnosed fracture could be initiated in an open model of care within three independent health care systems aided by a fracture liaison team and coordinated utilizing a cloud-based tool to track patients - To examine the barriers and challenges to this model, develop workable solutions for each system, and implement successful strategies to improve outcomes |

| **Study** | **Preventing future fractures: effectiveness of an orthogeriatric fracture liaison service compared to an outpatient fracture liaison service and the standard management in patients with hip fracture**  **Naranjo 2017** |
| --- | --- |
| Study type | Observational prospective study |
| Number of studies/ number of participants | N = 185 |
| Countries and settings | Two centers in Spain |
| Funding | None |
| Duration of study | Between 2014 and 2015 |
| Age, gender, ethnicity | Age(mean): outpatients: 79, inpatients HUGC:82, HUNS:82  Gender (% F): outpatients: 73%, HUGC: 80%, HUNS: 67%  Ethnicity: not reported |
| Patient characteristics | Adult patients aged 65 years or older with a hip fracture; patients with severe dementia and traumatic fractures were excluded, as were patients who died during the hospital stay |
| Intervention | Patients were admitted to an hospital with orthogeriatric standard care (HUNS Candelaria (N=105)) or in the hospital with fracture liaison service (outpatients HUGC Dr. Negrin (N=206), inpatients HUGC Dr. Negrin (N=80)) |
| Outcomes | - To compare the effectiveness of an orthogeriatric fracture liaison service, outpatient FLS, and the standard care after hip fractures in prevention of future fractures |

| **Study** | **Results after introduction of a hip fracture care pathway: comparison with usual care**  **Svenøy 2020** |
| --- | --- |
| Study type | Single center cohort study with historical controls |
| Number of studies/ number of participants | N = 443 |
| Countries and settings | Two centers in Spain |
| Funding | This work was funded by the hospital |
| Duration of study | Between 2015 and 2016 |
| Age, gender, ethnicity | Age(mean): intervention group: 81, control group: 82  Gender (% F): intervention group: 67%, control group: 77%  Ethnicity: not reported |
| Patient characteristics | Adult patients with hip fracture; patients with high-energy trauma and patients living in other hospital regions were excluded from the analyses |
| Intervention | Patients admitted in the orthopedic ward constituted the HFU (N= 276); patients from a previous trial in the hospital on hip fractures, the Oslo Orthogeriatric Trial, constituted the historical control group (N= 167) |
| Outcomes | - To provide better in-hospital care and thus improve outcome |

| **Study** | **The effectiveness of Police General Hospital’s fracture liaison service (PGH’s FLS) implementation after 5 years: A prospective cohort study**  **Amphansap 2020** |
| --- | --- |
| Study type | Prospective cohort study |
| Number of studies/ number of participants | N = 353 |
| Countries and settings | Police General Hospital, Bangkok, Thailand |
| Funding | This work was funded by the hospital |
| Duration of study | Between 2015 and 2016 |
| Age, gender, ethnicity | Age(%≤ 80, % >80): before project: 56.6%, 43.3%, after project:54.4%, 45.6%  Gender (% F): before project: 73.3%, after group: 73.65%  Ethnicity: not reported |
| Patient characteristics | Adult patients aged 50 years and older with low-energy fragility hip fracture; patients who had fractures due to high-energy trauma, bone tumors, and atypical femoral fractures were excluded |
| Intervention | Patients who participated in PGH’s FLS (after project (N=353)) were compared with a previous study, before the commencement of the FLS (before project (N=120)) |
| Outcomes | - To assess the effectiveness of fracture liaison service after 5-year implementation to close the secondary fracture care gap, ensuring that patients receive osteoporosis assessment, intervention, and treatment, therefore, reducing the fracture risk at police General Hospital (PGH) - secondary fragility fracture rates, and the mortality rates at 1-year follow-up after 5 years of PGH’s FLS implementation - type of treatment, time to surgery, length of hospital stay, the number of patients were treated for osteoporosis by medications at 1 year, post-injury ambulatory status at 1 year, post-injury number of falls at 1 year, the cause of loss of follow-up, and the cause of death |

| **Study** | **The impact of an orthogeriatric intervention in patients with fragility fractures: a cohort study**  **Abrahamsen 2019** |
| --- | --- |
| Study type | Prospective observational cohort study with a retrospective control |
| Number of studies/ number of participants | N = 591 |
| Countries and settings | Regional hospital serving a mixed rural and urban district in Denmark |
| Funding | None |
| Duration of study | Between 2014 and 2015 |
| Age, gender, ethnicity | Age(mean): orthogeriatric cohort: 80, historical cohort: 81  Gender (% F): orthogeriatric cohort: 78.2%, historical cohort: 77.2%  Ethnicity: not reported |
| Patient characteristics | Adult patients aged 65 years and older admitted to the orthogeriatric unit with a fragility fracture; patients were excluded if the fracture was cancer-related or caused by high-energy trauma, if the patient was operated on at another hospital, treated conservatively with no operation, or had been readmitted within the last month due to fracture-related complications |
| Intervention | Patients were admitted during two study periods: historical cohort (N=170) and orthogeriatric cohort (N=421) |
| Outcomes | - To assess the impact of an orthogeriatric intervention on postoperative complications and readmission among patients admitted due to and surgery treated for fragility fractures - To assess readmission rates with a notion of reduction |

| **Study** | **Orthogeriatric Trauma Unit Improves Patient Outcomes in Geriatric Hip Fracture Patients**  **Schuijt 2020** |
| --- | --- |
| Study type | Retrospective cohort study |
| Number of studies/ number of participants | N = 806 |
| Countries and settings | St. Antonius hospital, the Netherlands |
| Funding | None |
| Duration of study | Between January 2018 and December 2018 |
| Age, gender, ethnicity | Age(mean):intervention group: 85, control group: 85  Gender (% F): intervention group:71%, control group:73%  Ethnicity: not reported |
| Patient characteristics | Adult patients aged 70 years and older admitted to the ED with a hip fracture undergoing surgery were eligible; exclusion criteria were pathological hip fractures, total hip replacement surgery, and periprosthetic hip fractures |
| Intervention | The cohort was compared to a historical cohort before the implementation of the orthogeriatric trauma unit |
| Outcomes | - To evaluate outcomes of hip fracture patients admitted to the hospital before and after implementation of an orthogeriatric trauma unit - Postoperative complications - time spent at the ED, time to surgery, hospital length of stay, patient mortality, with a follow-up period of 1 year |

| **Study** | **Breaking the cycle of recurrent fracture: implementing the first fracture liaison service (FLS) in British Columbia, Canada**  **Singh 2019** |
| --- | --- |
| Study type | Controlled before-and-after study |
| Number of studies/ number of participants | N = 195 |
| Countries and settings | Canada |
| Funding | This study was supported by the Canadian Institutes of Health Research, Peace Arch Hospital Foundation, and the British Columbia Ministry of Health, Sonia Singh also obtained supplementary funding from Amgen Canada in the form of unrestricted grant-in-aid for the project |
| Duration of study | Between February 2015 and February 2016 |
| Age, gender, ethnicity | Age(mean):FLS group: 69.5, control group: 72.5  Gender (% F): FLS group:83.8%, control group:84.6%  Ethnicity: not reported |
| Patient characteristics | Adult patients aged 50 years and older with a low trauma fracture of the wrist, humerus, pelvis, hip or vertebrae. In addition, a small number of referrals from the orthopaedic surgeon were accepted; people were excluded from the study if they were under the age of 50 years, if there was a history of significant trauma or if they suffered from an underlying disease other than osteoporosis that leads to increased bone fragility. People were also excluded if they had significant cognitive dysfunction or insufficient English language skills to give informed consent and complete the study |
| Intervention | The intervention was an FLS program implemented at an orthopaedic outpatient clinic at Peace Arch Hospital in BC |
| Outcomes | - To evaluate the effectiveness of the first FLS program implemented in British Columbia, Canada - The percentage of all patients at high-risk to refracture, who achieved at least one of the following outcomes: started an osteoporosis medication, referred to an osteoporosis consultant or assessed for treatment change if they were already on osteoporosis medication at the time of the fracture - The rate of bone density testing, referral to fall prevention programs and change in health-related quality of life over 6 months |

| **Study** | **Does a fracture liaison service program minimize recurrent fragility fractures in the elderly with osteoporotic vertebral compression fractures?**  **Wasfie 2019** | |
| --- | --- | --- |
| Study type | Retrospective chart review | |
| Number of studies/ number of participants | N = 365 | |
| Countries and settings | USA | |
| Funding | Not reported | |
| Duration of study | Between January 2018 and December 2018 | |
| Age, gender, ethnicity | Age(mean):group A: 79, group B: 74.9  Gender (% F): group A:69%, group B:71%  Ethnicity: not reported | |
| Patient characteristics | Adult patients aged 50 years and older who presented to a local community hospital with a vertebral compression fracture who then followed up with the neurosurgery clinic. All patients included had not received any prior standard treatment for osteoporosis or osteopenia before the initial fracture | |
| Intervention | Patients were divided into two groups based on the time period of presentation to the hospital (group A N=150, group B N=215) | |
| Outcomes | - To evaluate outcomes of hip fracture patients admitted to the hospital before and after implementation of an orthogeriatric trauma unit - Postoperative complications - time spent at the ED, time to surgery, hospital length of stay, patient mortality, with a follow-up period of 1 year | |
| **Study** | **Clinical effectiveness of orthogeriatric and fracture liaison service models of care for hip fracture patients: population-based longitudinal study**  **Hawley 2016** |  |
| Study type | Population-based longitudinal study |  |
| Number of studies/ number of participants | N = 33152 |  |
| Countries and settings | UK hospital episode statistics database (HES), 11 acute hospitals in a region of England |  |
| Funding | This work was supported by the National Institutes of Health and Research (NIHR) Health Services and Delivery Research programme (HS&DR); and from the Oxford NIHR Musculoskeletal Biomedical Research Unit and Nuffield Orthopaedic Centre, University of Oxford |  |
| Duration of study | Between 2013 and 2015 |  |
| Age, gender, ethnicity | Age(mean):82.9  Gender (% F): 74.8%  Ethnicity: not reported |  |
| Patient characteristics | Adult patients aged 60 years admitted for a primary hip fracture; were excluded primary hip fractures admitted within the 12 months after an intervention |  |
| Intervention | Each hospital was analysed separately and acted as its own control in a before-after time-series design in which the appointment of an orthogeriatrician or set-up/expansion of an FLS was evaluated |  |
| Outcomes | - To evaluate orthogeriatric and nurse-led fracture liaison service (FLS) models of post-hip fracture care in terms of impact on mortality and second hip fracture |  |

# Supplemental Tables S4

## Quality evaluation

Observational (Newcastle-Ottawa Scale), randomized controlled trial (Cochrane Risk of Bias tool) and systematic reviews (Amstar-2)

Observational studies

|  | Selection | | | | Comparability | Outcome | | |  |
| --- | --- | --- | --- | --- | --- | --- | --- | --- | --- |
| Cohort study | Representativeness of the exposed cohort  (max 1) | Selection of the non exposed cohort  (max 1) | Ascertainment of exposure  (max 1) | Demonstration that outcome of interest was not present at start of study  (max 1) | Comparability of cohorts on the basis of the design or analysis  (max 2) | Assessment of outcome  (max 1) | Was follow-up long enough for outcomes to occur  (max 1) | Adequacy of follow-up of cohorts  (max 1) | tot |
| Wasfie 2019 | 1 | 1 | 1 | 1 | 2 | 1 | 1 | 1 | 9 |
| Vaculik 2017 | 1 | 1 | 1 | 1 | 0 | 1 | 1 | 1 | 7 |
| Shigemoto 2018 | 1 | 1 | 1 | 1 | 0 | 1 | 1 | 1 | 7 |
| Schuijt 2020 | 1 | 1 | 1 | 1 | 2 | 1 | 1 | 1 | 9 |
| Sanli 2019 | 1 | 1 | 1 | 1 | 2 | 1 | 1 | 1 | 9 |
| Rotman - Pikielny 2018 | 1 | 1 | 1 | 1 | 2 | 1 | 1 | 1 | 9 |
| Naranjo 2017 | 1 | 1 | 1 | 1 | 1 | 1 | 1 | 1 | 8 |
| Lamb 2017 | 1 | 1 | 1 | 1 | 0 | 1 | 1 | 1 | 7 |
| Inderjeeth 2018 | 1 | 1 | 1 | 1 | 1 | 1 | 1 | 1 | 8 |
| Hawley 2016 | 1 | 1 | 1 | 1 | 2 | 1 | 1 | 1 | 9 |
| Greenspan 2018 | 1 | 1 | 1 | 1 | 1 | 1 | 1 | 1 | 8 |
| Davidson 2017 | 1 | 1 | 1 | 1 | 1 | 1 | 1 | 1 | 8 |
| Coventry 2017 | 1 | 1 | 1 | 1 | 1 | 1 | 1 | 1 | 8 |
| Chan 2015 | 1 | 1 | 1 | 1 | 0 | 1 | 1 | 1 | 7 |
| Brañas 2018 | 1 | 1 | 1 | 1 | 0 | 1 | 1 | 1 | 7 |
| Beaupre 2020 | 1 | 1 | 1 | 1 | 0 | 1 | 1 | 1 | 7 |
| Beaton 2017 | 1 | 1 | 1 | 1 | 0 | 1 | 1 | 1 | 7 |
| Baroni 2019 | 1 | 1 | 1 | 1 | 2 | 1 | 1 | 1 | 9 |
| Bachour 2017 | 1 | 1 | 1 | 1 | 0 | 1 | 1 | 1 | 7 |
| Aubry-Rozier 2018 | 1 | 1 | 1 | 1 | 1 | 1 | 1 | 1 | 8 |
| Anighoro 2020 | 1 | 1 | 1 | 1 | 0 | 1 | 1 | 1 | 7 |
| Anderson 2017 | 1 | 1 | 1 | 1 | 0 | 1 | 1 | 1 | 7 |
| Amphansap 2020 | 1 | 1 | 1 | 1 | 0 | 1 | 1 | 1 | 7 |
| Abrahamsen 2019 | 1 | 1 | 1 | 1 | 1 | 1 | 1 | 1 | 8 |
| Sietsema 2018 | 1 | 1 | 1 | 0 | 0 | 1 | 1 | 0 | 5 |
| Singh 2019 | 1 | 1 | 1 | 0 | 1 | 1 | 1 | 0 | 6 |
| Sofie 2016 | 1 | 1 | 1 | 1 | 0 | 1 | 1 | 0 | 6 |
| Soong 2016 | 1 | 1 | 1 | 1 | 0 | 1 | 1 | 0 | 6 |
| Svenøy 2020 | 1 | 1 | 1 | 0 | 1 | 1 | 1 | 0 | 6 |

Randomized controlled trial

**Majumdar 2018**

| Bias | Authors' judgement | Support for judgement |
| --- | --- | --- |
| Random sequence generation  (selection bias) | LOW RISK | After agreeing to participate and providing written informed consent, participants were randomized via computer-generated randomization at a 1:1 group allocation with variable block size. |
| Allocation concealment  (selection bias) | LOW RISK | After agreeing to participate and providing written informed consent, participants were randomized via computer-generated randomization at a 1:1 group allocation with variable block size. |
| Blinding of participants and personnel (performance bias) | LOW RISK | Research nurses collected outcomes without knowledge of allocation status and investigators were blinded to both allocation status and outcomes. |
| Blinding of outcome assessment (detection bias) | LOW RISK | Research nurses collected outcomes without knowledge of allocation status and investigators were blinded to both allocation status and outcomes. |
| Incomplete outcome data  (attrition bias) | LOW RISK | The prespecified analysis was performed according to the intention-to-treat principle whereby participants were analyzed in the group to which they were allocated and those with missing data (n=11; 3%) were imputed as a patient who did not start bisphosphonate treatment (ie, missing=failure) for the primary outcome. |
| Selective reporting  (reporting bias) | LOW RISK | All outcomes mentioned in the earliest Version on record were analyzed and reported in the Results section (available on clinicaltrials.gov: NCT01401556) |
| Other bias | LOW RISK | Funding. This trial received funding from Alberta Innovates through a Partnership in Research and Innovation in the Healthcare System (PRIHS) grant and in-kind support from the Alberta Strategy for Patient-Oriented Research (SPOR) Support Unit. The funders take no responsibility for the conduct, results or opinions expressed in this manuscript.  Similarity at baseline. The groups were similar in sociodemographic and injury characteristics, health status, and previous bone health management at study entry. |

Systematic reviews

|  | **Ganda 2013** | **Bell 2014** | **Chang 2018** | **Wu 2018**  **doi: 10.1007/s00198-017-4370-z** | **Wu 2018**  **doi: 10.1016/j.bone.2018.03.018** | **Talevski 2019** |
| --- | --- | --- | --- | --- | --- | --- |
|  | 26 studies; search up to 2011 | 18 studies; search up to April 2013 | 24 studies; search up to February 2017 | 55 studies; search up to February 2017 | 75 studies; search up to February 2017 | 22 studies; up to July 25, 2018 |
| **OVERALL QUALITY** | **VERY LOW** | **VERY LOW** | **VERY LOW** | **VERY LOW** | **LOW** | **LOW** |
| 1-Question and inclusion | yes | yes | yes | yes | yes | yes |
| 2-Protocol | no | no | no | no | no | yes |
| 3-Study design | no | no | no | no | no | no |
| 4-Comprehensive search | yes | yes | yes | yes | yes | yes |
| 5-Study selection | yes | yes | yes | yes | yes | yes |
| 6-Data extraction | yes | partial yes | partial yes | partial yes | partial yes | yes |
| 7-Excluded studied justification | partial yes | partial yes | no | no | partial yes | partial yes |
| 8-Included studied details | yes | yes | yes | yes | yes | yes |
| 9-Risk of Bias | no | no | yes | partial yes | yes | yes |
| 10-Source of funding of included studies | no | no | no | no | no | no |
| 11-Appropriate statistical methods for analysis | no | N.A | N.A | no | yes | yes |
| 12-Rob on meta-analyses | no | N.A | N.A | yes | no | yes |
| 13-Rob on individual studies | no | no | no | yes | yes | yes |
| 14-Explanation for heterogeneity | yes | no | no | yes | yes | yes |
| 15-Publication bias | no | N.A | N.A | yes | yes | no |
| 16-Conflict of interest | yes | yes | yes | yes | yes | yes |
|  |  |  |  |  |  |  |
| **Tot Yes in critical flow** | 2 | 2 | 2 | 4 | 6 | 6 |
| **Tot No in critical flow** | 5 | 3 | 3 | 3 | 1 | 1 |
| **Tot No in critical weakness** | 3 | 3 | 3 | 2 | 3 | 2 |

# Supplemental Tables S5

## Summary of Findings, GRADE approach

**BMD TESTING RATE**

| **Certainty assessment** | | | | | | | **№ of patients** | | **Effect** | | **Certainty** | **Importance** |
| --- | --- | --- | --- | --- | --- | --- | --- | --- | --- | --- | --- | --- |
| **№ of studies** | **Study design** | **Risk of bias** | **Inconsistency** | **Indirectness** | **Imprecision** | **Other considerations** | **BMD testing** | **placebo** | **Relative (95% CI)** | **Absolute (95% CI)** |  |  |
| **after vs before (POOLED)** | | | | | | | | | | | | |
| 20 | observational studies | not serious | very serious^a^ | not serious | not serious | none | 10946/167274 (6.5%) | 5059/131123 (3.9%) | **RR 1.92** (1.44 to 2.55) | **35 more per 1.000** (from 17 more to 60 more) | ⨁◯◯◯ Very low | CRITICAL |
| **after vs before specialized model** | | | | | | | | | | | | |
| 11 | observational studies | not serious | very serious^a^ | not serious | not serious | none | 8887/40706 (21.8%) | 3982/25915 (15.4%) | **RR 2.29** (1.63 to 3.24) | **198 more per 1.000** (from 97 more to 344 more) | ⨁◯◯◯ Very low | CRITICAL |
| **after - after vs before FLS** | | | | | | | | | | | | |
| 9 | observational studies | not serious | very serious^a^ | not serious | not serious | none | 2059/126568 (1.6%) | 1077/105208 (1.0%) | **RR 1.54** (0.82 to 2.89) | **6 more per 1.000** (from 2 fewer to 19 more) | ⨁◯◯◯ Very low | CRITICAL |
| **FLS or specialized vs comparator model (POOLED)** | | | | | | | | | | | | |
| 9 | observational studies | not serious | very serious^a^ | not serious | not serious | none | 1486/2633 (56.4%) | 678/1410 (48.1%) | **RR 2.31** (1.40 to 3.82) | **630 more per 1.000** (from 192 more to 1.000 more) | ⨁◯◯◯ Very low | CRITICAL |
| **specialized model vs comparator model** | | | | | | | | | | | | |
| 6 | observational studies | not serious | very serious^a^ | not serious | not serious | none | 982/1786 (55.0%) | 502/871 (57.6%) | **RR 1.87** (1.15 to 3.04) | **501 more per 1.000** (from 86 more to 1.000 more) | ⨁◯◯◯ Very low | CRITICAL |
| **FLS vs comparator model** | | | | | | | | | | | | |
| 3 | observational studies | not serious | very serious^a^ | not serious | not serious | none | 504/847 (59.5%) | 176/539 (32.7%) | **RR 2.50** (1.05 to 5.93) | **490 more per 1.000** (from 16 more to 1.000 more) | ⨁◯◯◯ Very low | CRITICAL |
| **specialized model vs standard care** | | | | | | | | | | | | |
| 13 | observational studies | not serious | very serious^a^ | not serious | not serious | none | 1084/2860 (37.9%) | 445/2801 (15.9%) | **RR 2.45** (1.86 to 3.23) | **230 more per 1.000** (from 137 more to 354 more) | ⨁◯◯◯ Very low | CRITICAL |

**CI:** confidence interval; **RR:** risk ratio

Explanations

a. I2>75%

**TREATMENT INITIATION**

| **Certainty assessment** | | | | | | | **№ of patients** | | **Effect** | | **Certainty** | **Importance** |
| --- | --- | --- | --- | --- | --- | --- | --- | --- | --- | --- | --- | --- |
| **№ of studies** | **Study design** | **Risk of bias** | **Inconsistency** | **Indirectness** | **Imprecision** | **Other considerations** | **Treatment initiation** | **placebo** | **Relative (95% CI)** | **Absolute (95% CI)** |  |  |
| **after vs before (POOLED)** | | | | | | | | | | | | |
| 18 | observational studies | not serious | serious^a^ | not serious | not serious | none | 1805/5470 (33.0%) | 842/5573 (15.1%) | **RR 1.91** (1.58 to 2.29) | **137 more per 1.000** (from 88 more to 195 more) | ⨁◯◯◯ Very low | CRITICAL |
| **after vs before specialized model** | | | | | | | | | | | | |
| 13 | observational studies | not serious | serious^a^ | not serious | not serious | none | 647/2277 (28.4%) | 378/2334 (16.2%) | **RR 1.84** (1.43 to 2.38) | **136 more per 1.000** (from 70 more to 223 more) | ⨁◯◯◯ Very low | CRITICAL |
| **after vs before FLS** | | | | | | | | | | | | |
| 5 | observational studies | not serious | serious^a^ | not serious | not serious | none | 1158/3193 (36.3%) | 464/3239 (14.3%) | **RR 2.11** (1.66 to 2.70) | **159 more per 1.000** (from 95 more to 244 more) | ⨁◯◯◯ Very low | CRITICAL |
| **FLS or specialized model vs comparator model (POOLED)** | | | | | | | | | | | | |
| 10 | observational studies | not serious | very serious^a^ | not serious | not serious | none | 582/1898 (30.7%) | 241/1030 (23.4%) | **RR 1.38** (0.90 to 2.13) | **89 more per 1.000** (from 23 fewer to 264 more) | ⨁◯◯◯ Very low | CRITICAL |
| **specialized model vs comparator model** | | | | | | | | | | | | |
| 7 | observational studies | not serious | very serious^a^ | not serious | not serious | none | 428/1341 (31.9%) | 156/659 (23.7%) | **RR 1.26** (0.65 to 2.44) | **62 more per 1.000** (from 83 fewer to 341 more) | ⨁◯◯◯ Very low | CRITICAL |
| **FLS vs comparator model** | | | | | | | | | | | | |
| 3 | observational studies | not serious | not serious | not serious | not serious | none | 154/557 (27.6%) | 85/371 (22.9%) | **RR 1.60** (1.33 to 1.92) | **137 more per 1.000** (from 76 more to 211 more) | ⨁⨁◯◯ Low | CRITICAL |
| **FLS or specialized model vs standard care/non-attenders (POOLED)** | | | | | | | | | | | | |
| 15 | observational studies | not serious | very serious^a^ | not serious | not serious | none | 1244/3376 (36.8%) | 645/3189 (20.2%) | **RR 1.87** (1.50 to 2.32) | **176 more per 1.000** (from 101 more to 267 more) | ⨁◯◯◯ Very low | CRITICAL |
| **specialized model vs standard care** | | | | | | | | | | | | |
| 13 | observational studies | not serious | very serious^a^ | not serious | not serious | none | 1020/3069 (33.2%) | 507/2903 (17.5%) | **RR 1.91** (1.46 to 2.50) | **159 more per 1.000** (from 80 more to 262 more) | ⨁◯◯◯ Very low | CRITICAL |
| **FLS vs standard care** | | | | | | | | | | | | |
| 1 | observational studies | not serious | not serious | not serious | not serious | none | 50/93 (53.8%) | 14/66 (21.2%) | **RR 2.53** (1.53 to 4.19) | **325 more per 1.000** (from 112 more to 677 more) | ⨁⨁◯◯ Low | CRITICAL |
| **specialized model vs non-attenders** | | | | | | | | | | | | |
| 1 | observational studies | not serious | not serious | not serious | not serious | none | 174/214 (81.3%) | 124/220 (56.4%) | **RR 1.44** (1.26 to 1.65) | **248 more per 1.000** (from 147 more to 366 more) | ⨁⨁◯◯ Low | CRITICAL |

**CI:** confidence interval; **RR:** risk ratio

Explanations

1. I2>75%

**ADHERENCE**

| **Certainty assessment** | | | | | | | **№ of patients** | | **Effect** | | **Certainty** | **Importance** |
| --- | --- | --- | --- | --- | --- | --- | --- | --- | --- | --- | --- | --- |
| **№ of studies** | **Study design** | **Risk of bias** | **Inconsistency** | **Indirectness** | **Imprecision** | **Other considerations** | **Adherence** | **placebo** | **Relative (95% CI)** | **Absolute (95% CI)** |  |  |
| **after vs before (POOLED)** | | | | | | | | | | | | |
| 5 | observational studies | not serious | very serious^a^ | not serious | not serious | none | 16024/39813 (40.2%) | 11852/25051 (47.3%) | **RR 1.54** (1.03 to 2.31) | **255 more per 1.000** (from 14 more to 620 more) | ⨁◯◯◯ Very low | CRITICAL |
| **after vs before FLS** | | | | | | | | | | | | |
| 2 | observational studies | not serious | not serious | not serious | not serious | none | 149/218 (68.3%) | 57/175 (32.6%) | **RR 1.99** (1.58 to 2.49) | **322 more per 1.000** (from 189 more to 485 more) | ⨁⨁◯◯ Low | CRITICAL |
| **after vs before specialized model** | | | | | | | | | | | | |
| 3 | observational studies | not serious | very serious^a^ | not serious | not serious | none | 15875/39595 (40.1%) | 11795/24876 (47.4%) | **RR 1.29** (0.84 to 1.96) | **138 more per 1.000** (from 76 fewer to 455 more) | ⨁◯◯◯ Very low | CRITICAL |
| **FLS or specialized vs comparator model (POOLED)** | | | | | | | | | | | | |
| 3 | observational studies | not serious | not serious | not serious | not serious | none | 178/370 (48.1%) | 43/126 (34.1%) | **RR 1.41** (0.82 to 2.44) | **140 more per 1.000** (from 61 fewer to 491 more) | ⨁⨁◯◯ Low | CRITICAL |
| **specialized model vs comparator model** | | | | | | | | | | | | |
| 2 | observational studies | not serious | not serious | not serious | not serious | none | 156/325 (48.0%) | 20/77 (26.0%) | **RR 2.34** (0.57 to 9.69) | **348 more per 1.000** (from 112 fewer to 1.000 more) | ⨁⨁◯◯ Low | CRITICAL |
| **FLS vs comparator model** | | | | | | | | | | | | |
| 1 | observational studies | not serious | not serious | not serious | not serious | none | 22/45 (48.9%) | 23/49 (46.9%) | **RR 1.04** (0.68 to 1.59) | **19 more per 1.000** (from 150 fewer to 277 more) | ⨁⨁◯◯ Low | CRITICAL |
| **specialized model vs standard care** | | | | | | | | | | | | |
| 2 | observational studies | not serious | not serious | not serious | not serious | none | 335/362 (92.5%) | 272/334 (81.4%) | **RR 1.13** (1.01 to 1.26) | **106 more per 1.000** (from 8 more to 212 more) | ⨁⨁◯◯ Low | CRITICAL |

**CI:** confidence interval; **RR:** risk ratio

Explanations

a. I2>75%

**SUBSEQUENT FRACTURE**

| **Certainty assessment** | | | | | | | **№ of patients** | | **Effect** | | **Certainty** | **Importance** |
| --- | --- | --- | --- | --- | --- | --- | --- | --- | --- | --- | --- | --- |
| **№ of studies** | **Study design** | **Risk of bias** | **Inconsistency** | **Indirectness** | **Imprecision** | **Other considerations** | **Refracture** | **placebo** | **Relative (95% CI)** | **Absolute (95% CI)** |  |  |
| **after vs before (POOLED)** | | | | | | | | | | | | |
| 8 | observational studies | not serious | very serious^a^ | not serious | not serious | none | 787/127965 (0.6%) | 728/107044 (0.7%) | **RR 0.80** (0.49 to 1.33) | **1 fewer per 1.000** (from 3 fewer to 2 more) | ⨁◯◯◯ Very low | CRITICAL |
| **after vs before FLS** | | | | | | | | | | | | |
| 6 | observational studies | not serious | very serious^a^ | not serious | not serious | none | 659/126344 (0.5%) | 468/104818 (0.4%) | **RR 0.87** (0.44 to 1.72) | **1 fewer per 1.000** (from 3 fewer to 3 more) | ⨁◯◯◯ Very low | CRITICAL |
| **after vs before specialized model** | | | | | | | | | | | | |
| 2 | observational studies | not serious | not serious | not serious | not serious | none | 128/1621 (7.9%) | 260/2226 (11.7%) | **RR 0.65** (0.53 to 0.79) | **41 fewer per 1.000** (from 55 fewer to 25 fewer) | ⨁⨁◯◯ Low | CRITICAL |
| **Refracture FLS vs comparator** | | | | | | | | | | | | |
| 1 | observational studies | not serious | not serious | not serious | not serious | none | 18/332 (5.4%) | 11/214 (5.1%) | **RR 1.05** (0.51 to 2.19) | **3 more per 1.000** (from 25 fewer to 61 more) | ⨁⨁◯◯ Low | CRITICAL |
| **FLS or specialized vs standard care/non-attenders (POOLED)** | | | | | | | | | | | | |
| 7 | observational studies | not serious | serious^a^ | not serious | not serious | none | 216/2742 (7.9%) | 308/2939 (10.5%) | **RR 0.57** (0.37 to 0.87) | **45 fewer per 1.000** (from 66 fewer to 14 fewer) | ⨁◯◯◯ Very low | CRITICAL |
| **FLS vs standard care** | | | | | | | | | | | | |
| 2 | observational studies | not serious | not serious | not serious | not serious | none | 112/1614 (6.9%) | 138/1955 (7.1%) | **RR 0.75** (0.37 to 1.51) | **18 fewer per 1.000** (from 44 fewer to 36 more) | ⨁⨁◯◯ Low | CRITICAL |
| **specialized model vs standard care** | | | | | | | | | | | | |
| 2 | observational studies | not serious | not serious | not serious | not serious | none | 10/277 (3.6%) | 32/188 (17.0%) | **RR 0.21** (0.11 to 0.41) | **134 fewer per 1.000** (from 151 fewer to 100 fewer) | ⨁⨁◯◯ Low | CRITICAL |
| **FLS vs non-attenders** | | | | | | | | | | | | |
| 2 | observational studies | not serious | not serious | not serious | not serious | none | 80/637 (12.6%) | 97/576 (16.8%) | **RR 0.79** (0.55 to 1.14) | **35 fewer per 1.000** (from 76 fewer to 24 more) | ⨁⨁◯◯ Low | CRITICAL |
| **specialized model vs non-attenders** | | | | | | | | | | | | |
| 1 | observational studies | not serious | not serious | not serious | not serious | none | 14/214 (6.5%) | 41/220 (18.6%) | **RR 0.35** (0.20 to 0.62) | **121 fewer per 1.000** (from 149 fewer to 71 fewer) | ⨁⨁◯◯ Low | CRITICAL |

**CI:** confidence interval; **RR:** risk ratio

Explanations

a. I2>75%

**MORTALITY**

| **Certainty assessment** | | | | | | | **№ of patients** | | **Effect** | | **Certainty** | **Importance** |
| --- | --- | --- | --- | --- | --- | --- | --- | --- | --- | --- | --- | --- |
| **№ of studies** | **Study design** | **Risk of bias** | **Inconsistency** | **Indirectness** | **Imprecision** | **Other considerations** | **Mortality** | **placebo** | **Relative (95% CI)** | **Absolute (95% CI)** |  |  |
| **after vs before (POOLED)** | | | | | | | | | | | | |
| 17 | observational studies | not serious | serious^a^ | not serious | not serious | none | 930/8004 (11.6%) | 1169/8127 (14.4%) | **RR 0.81** (0.66 to 1.00) | **27 fewer per 1.000** (from 49 fewer to 0 fewer) | ⨁◯◯◯ Very low | CRITICAL |
| **after vs before FLS** | | | | | | | | | | | | |
| 5 | observational studies | not serious | not serious | not serious | not serious | none | 447/3336 (13.4%) | 450/3282 (13.7%) | **RR 1.00** (0.80 to 1.25) | **0 fewer per 1.000** (from 27 fewer to 34 more) | ⨁⨁◯◯ Low | CRITICAL |
| **after vs before specialized model** | | | | | | | | | | | | |
| 12 | observational studies | not serious | serious^a^ | not serious | not serious | none | 483/4668 (10.3%) | 719/4845 (14.8%) | **RR 0.72** (0.54 to 0.95) | **42 fewer per 1.000** (from 68 fewer to 7 fewer) | ⨁◯◯◯ Very low | CRITICAL |
| **FLS or specialized model vs comparator model (POOLED)** | | | | | | | | | | | | |
| 3 | observational studies | not serious | not serious | not serious | not serious | none | 17/522 (3.3%) | 28/428 (6.5%) | **RR 0.52** (0.26 to 1.03) | **31 fewer per 1.000** (from 48 fewer to 2 more) | ⨁⨁◯◯ Low | CRITICAL |
| **FLS vs comparator model** | | | | | | | | | | | | |
| 1 | observational studies | not serious | not serious | not serious | not serious | none | 7/332 (2.1%) | 15/214 (7.0%) | **RR 0.30** (0.12 to 0.73) | **49 fewer per 1.000** (from 62 fewer to 19 fewer) | ⨁⨁◯◯ Low | CRITICAL |
| **specialized model vs comparator model** | | | | | | | | | | | | |
| 2 | observational studies | not serious | not serious | not serious | not serious | none | 10/190 (5.3%) | 13/214 (6.1%) | **RR 0.79** (0.35 to 1.79) | **13 fewer per 1.000** (from 39 fewer to 48 more) | ⨁⨁◯◯ Low | CRITICAL |
| **FLS or specialized vs standard care/non-attenders (POOLED)** | | | | | | | | | | | | |
| 9 | observational studies | not serious | serious^a^ | not serious | not serious | none | 404/2924 (13.8%) | 508/3388 (15.0%) | **RR 0.68** (0.48 to 0.96) | **48 fewer per 1.000** (from 78 fewer to 6 fewer) | ⨁◯◯◯ Very low | CRITICAL |
| **FLS vs standard care** | | | | | | | | | | | | |
| 2 | observational studies | not serious | very serious^a^ | not serious | not serious | none | 182/1618 (11.2%) | 282/2158 (13.1%) | **RR 0.72** (0.40 to 1.29) | **37 fewer per 1.000** (from 78 fewer to 38 more) | ⨁◯◯◯ Very low | CRITICAL |
| **specialized model vs standard care** | | | | | | | | | | | | |
| 4 | observational studies | not serious | not serious | not serious | not serious | none | 16/455 (3.5%) | 26/434 (6.0%) | **RR 0.54** (0.21 to 1.37) | **28 fewer per 1.000** (from 47 fewer to 22 more) | ⨁⨁◯◯ Low | CRITICAL |
| **FLS vs non-attenders** | | | | | | | | | | | | |
| 2 | observational studies | not serious | very serious^a^ | not serious | not serious | none | 200/637 (31.4%) | 166/576 (28.8%) | **RR 0.99** (0.60 to 1.63) | **3 fewer per 1.000** (from 115 fewer to 182 more) | ⨁◯◯◯ Very low | CRITICAL |
| **specialized model vs non-attenders** | | | | | | | | | | | | |
| 1 | observational studies | not serious | not serious | not serious | not serious | none | 6/214 (2.8%) | 34/220 (15.5%) | **RR 0.18** (0.08 to 0.42) | **127 fewer per 1.000** (from 142 fewer to 90 fewer) | ⨁⨁◯◯ Low | CRITICAL |

**CI:** confidence interval; **RR:** risk ratio

Explanations

a. I2>75%

# Supplemental Tables S6

## Summary Results

|  | | **Number of studies**  **(N of RCT)** | **Number and % of patients** | **Number and % of patients in RCT** | **Follow-up**  **(min-max. months)** | **Relative Risk**  **(95% CI), I^2^** |
| --- | --- | --- | --- | --- | --- | --- |
|  | **Evaluation of BMD testing rate** | | | | |  |
|  | a) after vs before specialized or FLS model implementation | | | | |  |
| Total | | 20 (0) | 167274/131123 (56.1/43.9) | 0/0 | 3-36 | 1.92 (1.44-2.55); I^2^= 98% |
| After vs before specialized model | | 11 (0) | 40706/25915 (61.1/38.9) | 0/0 | 3-17 | 2.29 (1.63-3.24); I^2^= 97% |
| After vs before FLS model | | 9 (0) | 126568/105208 (54.6/45.4) | 0/0 | 6-36 | 1.54 (0.82-2.89); I^2^= 99% |
|  | b) specialized or FLS model vs comparator model | | | | |  |
| Total | | 9 (2) | 2633/1410 (65.1/34.9) | 201/206 (49.4/50.6) | 3-24 | 2.31 (1.40-3.82); I^2^= 97% |
| Specialized model vs comparator model | | 6 (1) | 1786/871 (67.2/32.8) | 21/25 (45.7/54.3) | 3-24 | 1.87 (1.15-3.04); I^2^= 89% |
| FLS model vs comparator model | | 3 (1) | 847/539 (61.1/38.9) | 180/181 (49.9/50.1) | 6-12 | 2.50 (1.05-5.93); I^2^= 97% |
|  | c) specialized model vs standard care | | | | |  |
| Specialized model vs standard care | | 13 (10) | 2860/2801 (50.5/49.5) | 2543/2500 (50.4/49.6) | 3-12 | 2.45 (1.86-3.23); I^2^= 88% |
|  | **Evaluation of antiosteoporotic initiation** | | | | |  |
|  | a) after vs before specialized or FLS model implementation | | | | |  |
| Total | | 18 (0) | 5470/5573 (49.5/50.5) | 0/0 | 3-72 | 1.91 (1.58-2.29); I^2^= 78% |
| After vs before specialized model | | 13 (0) | 2277/2334 (49.4/50.6) | 0/0 | 3-72 | 1.84 (1.43-2.38); I^2^= 77% |
| After vs before FLS model | | 5 (0) | 3193/3239 (49.6/50.4) | 0/0 | 6-24 | 2.11 (1.66-2.70); I^2^= 70% |
|  | b) specialized or FLS model vs comparator model | | | | |  |
| Total | | 10 (3) | 1898/1030 (64.8/35.2) | 408/393 (50.9/49.1) | 6-48 | 1.38 (0.90-2.13); I^2^= 91% |
| Specialized model vs comparator model | | 7 (2) | 1341/659 (67.1/33.0) | 228/212 (51.8/48.2) | 6-48 | 1.26 (0.65-2.44); I^2^= 93% |
| FLS vs comparator model | | 3 (1) | 154/371 (29.3/70.7) | 180/181 (49.9/50.1) | 6 | 1.60 (1.33-1.92); I^2^= 0% |
|  | c) specialized or FLS model vs standard care/non-attenders | | | | |  |
| Total | | 15 (10) | 3376/3189 (51.4/48.6) | 2543/2500 (50.4/49.6) | 3-48 | 1.87 (1.50-2.32); I^2^= 71.8% |
| Specialized model vs standard care | | 13 (10) | 3069/2903 (51.4/48.6) | 2543/2500 (50.4/49.6) | 3-48 | 1.91 (1.46-2.50); I^2^= 90% |
| FLS model vs standard care | | 1 (0) | 93/66 (58.5/41.5) | 0/0 | 6 | 2.53 (1.53-4.19); na |
| Specialized model vs non-attenders | | 1 (0) | 214/220 (49.3/50.7) | 0/0 | 24 | 1.44 (1.26-1.65); na |
|  | **Evaluation of antiosteoporotic adherence** | | | | |  |
|  | a) after vs before specialized or FLS model implementation | | | | |  |
| Total | | 5 (0) | 39813/25051 (61.4/38.6) | 0/0 | 12-24 | 1.54 (1.03-2.31); I^2^= 96% |
| After vs before specialized model | | 3 (0) | 39595/24876 (61.4/38.6) | 0/0 | 12 | 1.29 (0.84-1.96); I^2^= 96% |
| After vs before FLS model | | 2 (0) | 218/175 (55.5/44.5) | 0/0 | 12-24 | 1.99 (1.58-2.49); I^2^= 0% |
|  | b) FLS or specialized model vs comparator model | | | | |  |
| Total | | 3 (1) | 1898/126 (93.8/6.2) | 408/48 (89.5/10.5) | 6-48 | 1.41 (0.82-2.44); I^2^= 59% |
| Specialized model vs comparator model | | 2 (0) | 325/77 (80.8/19.2) | 0/0 | 6-48 | 2.34 (0.57-9.69); I^2^= 40% |
| FLS model vs comparator model | | 1 (1) | 45/49 (47.9/52.1) | 45/49 (47.9/52.1) | 24 | 1.04 (0.68-1.59); na |
|  | c) specialized model vs standard care | | | | |  |
| Specialized model vs standard care | | 2 (1) | 362/334 (52.0/48.0) | 137/135 (50.4/49.6) | 6 | 1.31 (1.01-1.26); I^2^= 74% |
|  | **Evaluation of the risk of subsequent fracture** | | | | |  |
|  | a) after vs before specialized or FLS model implementation | | | | |  |
| Total | | 8 (0) | 127965/107044 (54.5/45.5) | 0/0 | 6-72 | 0.80 (0.49-1.33); I^2^= 95% |
| After vs before specialized model | | 2 (0) | 1621/2226 (42.1/57.9) | 0/0 | 24-72 | 0.65 (0.53-0.79); I^2^= 0% |
| After vs before FLS model | | 6 (0) | 126344/104818 (54.7/45.3) | 0/0 | 6-36 | 0.87 (0.44-1.72); I^2^= 95% |
|  | b) FLS model vs comparator model | | | | |  |
| FLS model vs comparator model | | 1 (0) | 332/214 (60.8/39.2) | 0/0 | 12 | 1.05 (0.51-2.19); na |
|  | c) specialized or FLS model vs standard care/non-attenders | | | | |  |
| Total | | 7 (1) | 2742/2939 (48.3/51.7) | 31/31 (50.0/50.0) | 3-48 | 0.57 (0.37-0.87); I^2^= 79% |
| Specialized model vs standard care | | 2 (1) | 277/188 (59.6/40.4) | 31/31 (50.0/50.0) | 6-48 | 0.21 (0.11-0.41); I^2^= 0% |
| FLS model vs standard care | | 2 (0) | 1614/1955 (45.2/54.8) | 0/0 | 12-24 | 0.75 (0.37-1.51); I^2^= 68% |
| Specialized model vs non-attenders | | 1 (0) | 214/220 (49.3/50.7) | 0/0 | 24 | 0.35 (0.20-0.62); na |
| FLS model vs non-attenders | | 1 (0) | 637/576 (52.5/47.5) | 0/0 | 24-36 | 0.79 (0.55-1.14); I^2^= 38% |
|  | **Evaluation of the risk of mortality** | | | | |  |
|  | a) after vs before the specialized or FLS model implementation | | | | |  |
| Total | | 17 (0) | 8004/8127 (49.6/50.4) | 0/0 | 1-72 | 0.81 (0.66-1.00); I^2^= 75% |
| After vs before specialized model | | 12 (0) | 4668/4845 (49.1/50.9) | 0/0 | 1-72 | 0.72 (0.54-0.95); I^2^= 75% |
| After vs before FLS model | | 5 (0) | 3336/3282 (50.4/49.6) | 0/0 | 12-36 | 1.00 (0.80-1.25); I^2^= 34% |
|  | b) specialized or FLS model vs comparator model | | | | |  |
| Total | | 3 (0) | 522/428 (54.9/45.1) | 0/0 | 12-48 | 0.52 (0.26-1.03); I^2^= 21% |
| Specialized model vs comparator model | | 2 (0) | 190/214 (47.0/53.0) | 0/0 | 12-48 | 0.79 (0.35-1.79); I^2^= 0% |
| FLS model vs comparator model | | 1 (0) | 332/214 (60.8/39.2) | 0/0 | 12 | 0.30 (0.12-0.73); na |
|  | c) specialized or FLS model vs standard care/non-attenders | | | | |  |
| Total | | 9 (3) | 2924/3388 (46.3/53.7) | 323/330 (49.5/50.5) | 6-36 | 0.68 (0.48-0.96); I^2^= 78% |
| Specialized model vs standard care | | 4 (3) | 455/434 (51.2/48.8) | 323/330 (49.5/50.5) | 6-12 | 0.54 (0.21-1.37); I^2^= 30% |
| FLS model vs standard care | | 2 (0) | 1618/2158 (42.8/57.2) | 0/0 | 12-24 | 0.72 (0.40-1.29); I^2^= 80% |
| Specialized model vs non-attenders | | 1 (0) | 214/220 (49.3/50.7) | 0/0 | 24 | 0.18 (0.08-0.42); na |
| FLS model vs non-attenders | | 2 (0) | 637/576 (52.5/47.5) | 0/0 | 24-36 | 0.99 (0.60-1.63); I^2^= 83% |

# Supplemental Figure S1

## Funnel plot and Egger’s test

Publication bias in the studies on 1) BMD testing rate, 2) antiosteoporotic initiation, or 3) mortality rate evaluation.

Abbreviations: RR, relative risk; SE, standard error

1a) after vs before the specialized or Fracture liaison service (FLS) model implementation


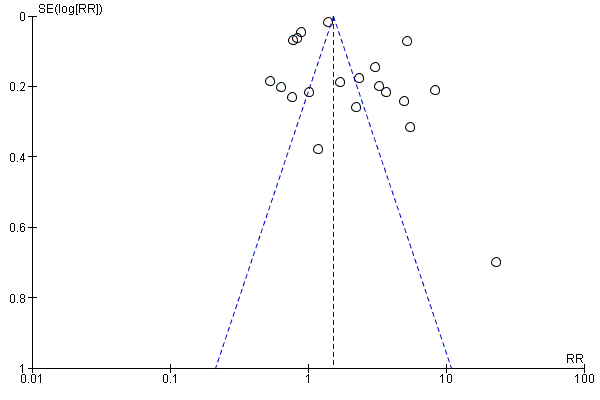


p= 0.29

1b) specialized model vs standard care


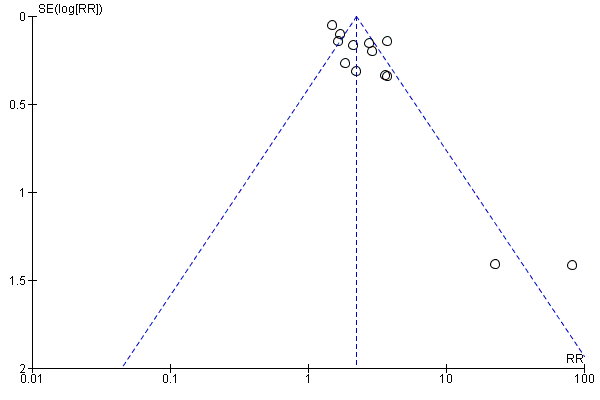


p= 0.69

2a) after vs before the specialized or Fracture liaison service (FLS) model implementation


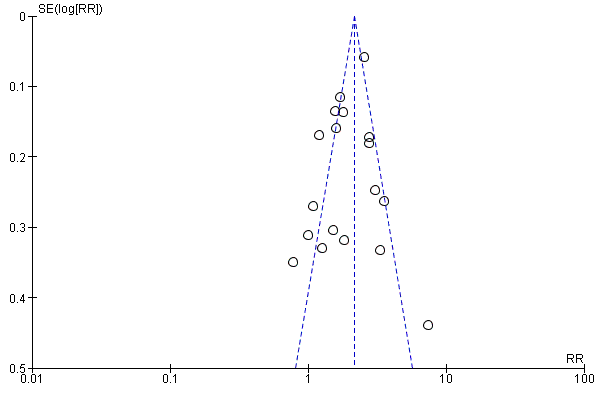


p= 0.46

2b) specialized or FLS model vs comparator model


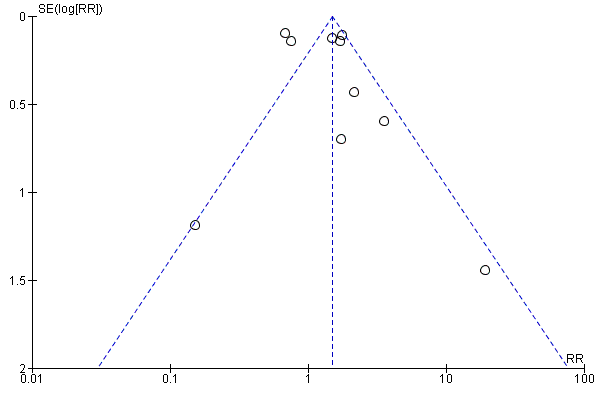


p= 0.55

2c) specialized model or FLS vs standard care/non-attenders


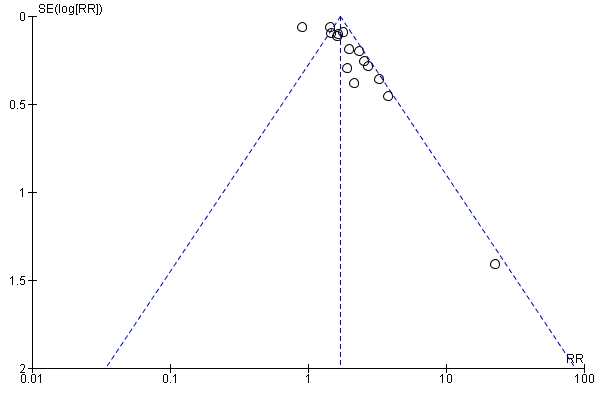


p= 0.67

3a) after vs before the specialized or Fracture liaison service (FLS) model implementation


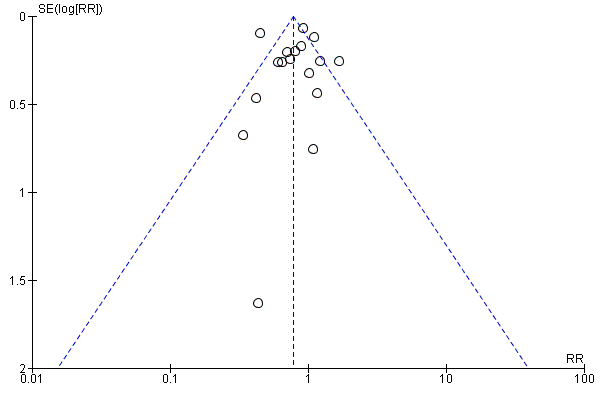


p= 0.41

# Supplemental Figure S2

## BMD testing rate, RCT studies

Evaluation of the BMD testing rate in the Fracture liaison service group or specialized model compared to comparator model or standard care among the randomized controlled study.

Squares represent study-specific relative risk estimates (size of the square reflects the study-specific statistical weight, that is, the inverse of the variance); horizontal lines represent 95% CIs; diamonds represent summary relative risk estimates with corresponding 95% CIs; p values are from testing for heterogeneity between study-specific estimates. Abbreviations: CI confidence interval, RR relative risk


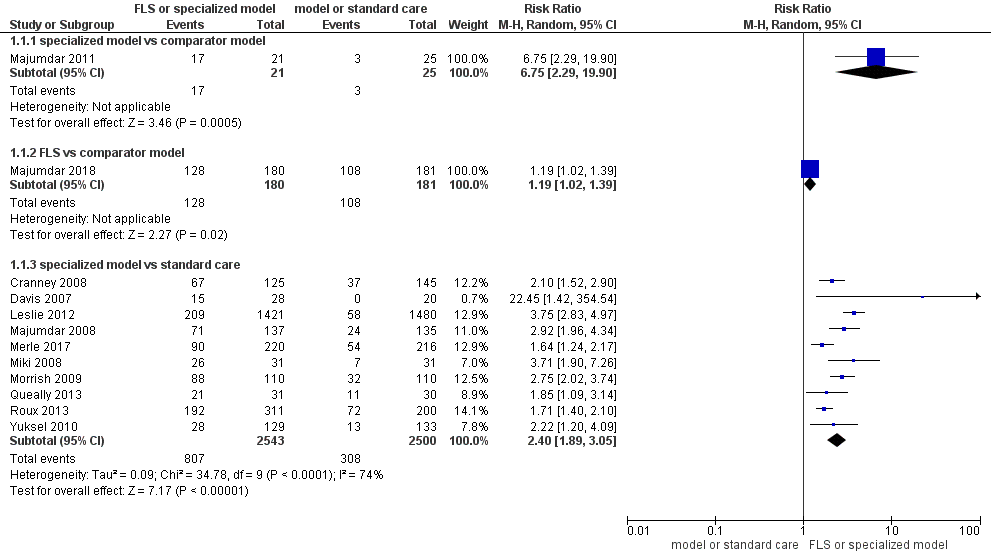


# Supplemental Figure S3

## Antiosteoporotic initiation, RCT studies

Evaluation of the antiosteoporotic initiation in the Fracture liaison service group or specialized model compared to comparator model or standard care among the randomized controlled study.

Squares represent study-specific relative risk estimates (size of the square reflects the study-specific statistical weight, that is, the inverse of the variance); horizontal lines represent 95% CIs; diamonds represent summary relative risk estimates with corresponding 95% CIs; p values are from testing for heterogeneity between study-specific estimates. Asterisk indicates randomized controlled studies. Abbreviations: CI confidence interval, RR relative risk


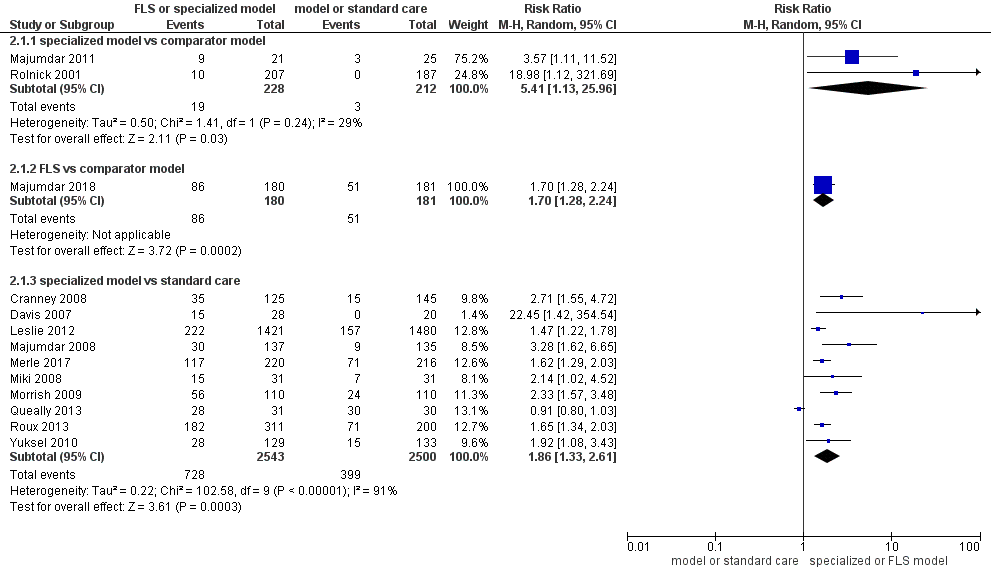


# Supplemental Figure S4

## Antiosteoporotic adherence, RCT studies

Evaluation of the adherence to antiosteoporotic treatment in the Fracture liaison service group or specialized model compared to comparator model or standard care among the randomized controlled study.

Squares represent study-specific relative risk estimates (size of the square reflects the study-specific statistical weight, that is, the inverse of the variance); horizontal lines represent 95% CIs; diamonds represent summary relative risk estimates with corresponding 95% CIs; p values are from testing for heterogeneity between study-specific estimates. Abbreviations: CI confidence interval, RR relative risk


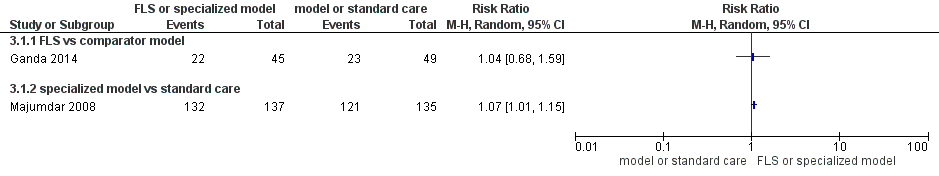


# Supplemental Figure S5

## Refracture risk, RCT studies

Evaluation of the refracture risk in the specialized model compared to standard care among the randomized controlled study.

Squares represent study-specific relative risk estimates (size of the square reflects the study-specific statistical weight, that is, the inverse of the variance); horizontal lines represent 95% CIs; diamonds represent summary relative risk estimates with corresponding 95% CIs; p values are from testing for heterogeneity between study-specific estimates. Abbreviations: CI confidence interval, RR relative risk


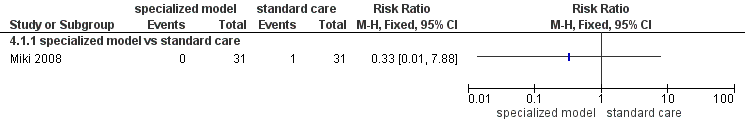


# Supplemental Figure S6

## Mortality risk, RCT studies

Evaluation of the mortality risk in the specialized model compared to standard care among the randomized controlled study.

Squares represent study-specific relative risk estimates (size of the square reflects the study-specific statistical weight, that is, the inverse of the variance); horizontal lines represent 95% CIs; diamonds represent summary relative risk estimates with corresponding 95% CIs; p values are from testing for heterogeneity between study-specific estimates. Abbreviations: CI confidence interval, RR relative risk


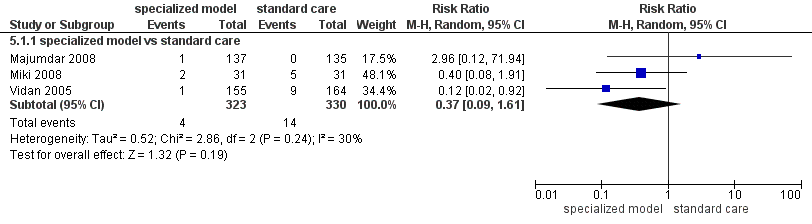


# Complete list of experts involved

**Fragility Fracture Team**

*Giovanni Adami Member of SIOMMMS - Società Italiana di Osteoporosi del metabolismo minerale e delle malattie dello scheletro*

*Rosaria Alvaro Associate Professor in Nursing Sciences – University of Rome Tor Vergata*

*Annalisa Biffi Department of Statistics and Quantitative Methods; Healthcare Research & Pharmacoepidemiology Interuniversity Center, University of Milan-Bicocca*

*Riccardo Bogini General Pratictioner at USL Umbria*

*Maria Luisa Brandi Full Professor of Endocrinology and Metabolic Bone Diseases - University of Florence; Director of the Regional Referral Center for Hereditary Endocrine Tumors; Director Clinical Unit on Metabolic Bone Disorders - University Hospital of Florence; President of FIRMO - Fondazione Italiana Ricerca sulle Malattie dell’Osso*

*Achille Patrizio Caputi Emeritus Professor of Pharmacology; University of Messina*

*Luisella Cianferotti Associate Professor of Endocrinology, University of Florence; member of FIRMO - Fondazione Italiana Ricerca sulle Malattie dell’Osso*

*Giovanni Corrao Full Professor of Medical Statistics – University of Milan-Bicocca; Director of Healthcare Research & Pharmacoepidemiology Interuniversity Center*

*Bruno Frediani Full Professor of Rheumatology; Director of the School of Specialization in Rheumatology - University of Siena; Director of the Complex Operational Unit in Rheumatology; Director of the Department of Medical Sciences*

*Davide Gatti Associate Professor of Rheumatology – University of Verona; President of the Scientific Committee of the ASITOI - Associazione Italiana Osteogenesi Imperfetta; Coordinator of the Guideline Commission SIOMMMS - Società Italiana di Osteoporosi del metabolismo minerale e delle malattie dello scheletro*

*Stefano Gonnelli Full Professor of Internal Medicine and Director of the School of Specialization in Iternal Medicine - University of Siena*

*Giovanni Iolascon Full Professor of Physical and Rehabilitation Medicine – University of Campania “Luigi Vanvitelli”*

*Andrea Lenzi Full Professor of Endocrinology - University of Rome La Sapienza; member of SIE – Società italiana di Endocrinologia*

*Salvatore Leone Member of AMICI Onlus - Associazione nazionale per le Malattie Infiammatorie Croniche dell'Intestino*

*Raffaella Michieli National Secretary SIMG – Società italiana di medicina generale e delle cure primarie*

*Silvia Migliaccio Member of SIE – Società italiana di Endocrinologia; Associate Professor - University of Rome Foro Italico*

*Tiziana Nicoletti Manager of CnAMC - Coordinamento nazionale delle Associazioni dei Malati Cronici e rari di Cittadinanzattiva*

*Marco Paoletta Member of SIMFER - Società Italiana di Medicina Fisica e Riabilitativa*

*Annalisa Pennini Member of FNOPI - Federazione Nazionale degli Ordini delle Professioni Infermieristiche per il progetto Fratture da Fragilità*

*Eleonora Piccirilli Department of Orthopedics and Traumatology, University of Rome Tor Vergata*

*Gloria Porcu Department of Statistics and Quantitative Methods; Healthcare Research & Pharmacoepidemiology Interuniversity Center, University of Milan-Bicocca*

*Raffaella Ronco Department of Statistics and Quantitative Methods; Healthcare Research & Pharmacoepidemiology Interuniversity Center, University of Milan-Bicocca*

*Maurizio Rossini Full Professor of Rheumatology; President of SIOMMMS - Società Italiana dell'Osteoporosi, del Metabolismo Minerale e delle Malattie dello Scheletro; Member of SIR - Società Italiana di Reumatologia*

*Umberto Tarantino Full Professor of Diseases of the Locomotor System – University of Rome; Member of SIOT – Società italiana di ortopedia e traumatologia*
